# Nucleosome composition regulates the histone H3 tail conformational ensemble and accessibility

**DOI:** 10.1093/nar/gkab246

**Published:** 2021-04-15

**Authors:** Emma A Morrison, Lokesh Baweja, Michael G Poirier, Jeff Wereszczynski, Catherine A Musselman

**Affiliations:** Department of Biochemistry, Carver College of Medicine, University of Iowa, Iowa City, IA, USA; Department of Biochemistry, Medical College of Wisconsin, Milwaukee, WI, USA; Department of Physics, Illinois Institute of Technology, Chicago, IL, USA; Center for Molecular Study of Condensed Soft Matter, Illinois Institute of Technology, Chicago, IL, USA; Department of Physics, Biophysics Graduate Program, Ohio State Biochemistry Graduate Program, and Department of Chemistry and Biochemistry, The Ohio State University, Columbus, OH, USA; Department of Physics, Illinois Institute of Technology, Chicago, IL, USA; Center for Molecular Study of Condensed Soft Matter, Illinois Institute of Technology, Chicago, IL, USA; Department of Biochemistry, Carver College of Medicine, University of Iowa, Iowa City, IA, USA; Department of Biochemistry and Molecular Genetics, University of Colorado Anschutz Medical Campus, Aurora, CO, USA

## Abstract

Hexasomes and tetrasomes are intermediates in nucleosome assembly and disassembly. Their formation is promoted by histone chaperones, ATP-dependent remodelers, and RNA polymerase II. In addition, hexasomes are maintained in transcribed genes and could be an important regulatory factor. While nucleosome composition has been shown to affect the structure and accessibility of DNA, its influence on histone tails is largely unknown. Here, we investigate the conformational dynamics of the H3 tail in the hexasome and tetrasome. Using a combination of NMR spectroscopy, MD simulations, and trypsin proteolysis, we find that the conformational ensemble of the H3 tail is regulated by nucleosome composition. As has been found for the nucleosome, the H3 tails bind robustly to DNA within the hexasome and tetrasome, but upon loss of the H2A/H2B dimer, we determined that the adjacent H3 tail has an altered conformational ensemble, increase in dynamics, and increase in accessibility. Similar to observations of DNA dynamics, this is seen to be asymmetric in the hexasome. Our results indicate that nucleosome composition has the potential to regulate chromatin signaling and ultimately help shape the chromatin landscape.

## INTRODUCTION

The eukaryotic genome is packaged into the cell nucleus in the form of chromatin. The basic subunit of chromatin is the nucleosome, a complex of histone proteins and DNA. The canonical nucleosome core particle consists of ∼147 base-pairs (bp) of DNA wrapped around an octamer containing one H3/H4 tetramer and two H2A/H2B dimers. In addition to this canonical species, sub-nucleosomal species, which contain fewer than eight histones, have been identified. These include the hexasome and tetrasome, which are lacking one or both H2A/H2B dimers, respectively (Supplementary Figure S1A).

For some time, these species have been studied *in vitro* and have been suggested to play a role in cellular processes such as transcription (reviewed in (1)). Hexasomes and tetrasomes are intermediates in chaperone-mediated and salt-dependent nucleosome assembly/disassembly (2–9). In addition, hexasomes form during transcription and ATP-dependent remodeling of nucleosomes (10–13). Furthermore, the presence of hexasomes versus nucleosomes differentially affects the activity of RNA polymerase II (14) and the CHD1 chromatin remodeler (15,16), supporting a regulatory role for sub-nucleosomes. Recently, these species have been observed *in vivo* (17,18). It has been suggested that hexasomes exist as stable species near transcription start sites and may be an important regulatory factor (17,18).

A number of structural and biophysical studies have allowed for characterization of these species (6,19–26). These studies have revealed that the histone core composition influences the DNA conformation and accessibility. Loss of an H2A/H2B dimer leads to unwrapping of ∼30–40 bp of DNA, which alters accessibility to digestion by endonuclease and transcription factor binding (15,19,22,27). Notably, while the nucleosome and tetrasome are structurally pseudo-symmetric particles, the hexasome is structurally asymmetric both in the histone core and the associated DNA wrapping (1,6,15,20,22,23,27). Intriguingly, DNA unwrapping and dimer loss have been observed to be asymmetric both *in vitro* and *in vivo* (18,27)*. In vitro* studies reveal a dependence on DNA sequence, and *in vivo* this is correlated with transcriptional activity (27). It has been proposed that this asymmetry may be important in reinforcing directional activity of RNA polymerase and chromatin remodelers (14,15,17,18).

A number of studies have indicated that the H3 tails can associate with DNA in the context of the nucleosome (25,28–36). This occlusion of the tails has further been observed to restrict access to histone tail binding domains (30,35–37). Using nuclear magnetic resonance (NMR) spectroscopy and molecular dynamics (MD) simulations, we recently proposed a structural model of the H3 tails in which they adopt a ‘fuzzy’ complex with DNA (38–42), interacting robustly but adopting a heterogenous and dynamic ensemble of DNA-bound states (35). This model suggests that chromatin signaling events could be regulated by modulating the DNA-bound conformational ensemble of the H3 tails. In the canonical nucleosome, the H3 tails protrude from between the two gyres of DNA near the entry/exit sites. Our previous MD simulations as well as cross-linking data indicate that the tails form interactions with both gyres (35,43). Thus, the loss of one or both H2A/H2B dimers and subsequent DNA unwrapping is predicted to significantly alter the conformational ensemble and possibly accessibility of the H3 tails.

Here, using a combination of NMR spectroscopy, MD simulations, and proteolysis assays we show that the H3 tails adopt distinct conformational ensembles in nucleosome, hexasome, and tetrasome. Our results indicate that loss of H2A/H2B dimer(s) leads to an increase in the conformational dynamics of the H3 tail and accessibility to binding. Similar to the DNA dynamics, in the hexasome these effects are seen to be asymmetric. Together, these data suggest that conversion between nucleosome, hexasome, and tetrasome may modulate chromatin signaling at the histone tails and that this could function synergistically with concomitant changes in DNA accessibility.

## MATERIALS AND METHODS

### Histone and DNA purification

Histones and 147 bp Widom 601 DNA were expressed/amplified and purified as described in (35,44).

### Mass spectrometry on histone samples

Electrospray ionization mass spectrometry was used to analyze the histones to confirm that there was no carbamylation as described in (35).

### Generation of nucleosomes and subnucleosomes

Nucleosomes were largely reconstituted as described in (44). Nucleosome reconstitutions were prepared with two variations—either (i) by refolding octamer with equimolar ratios of the histones H2A, H2B, H3 and H4 or (ii) by refolding tetramer (with equimolar ratios of H3 and H4) and dimer (with equimolar ratios of H2A and H2B) separately. Then, either (i) the octamer was mixed with 601 DNA at a 1:1 molar ratio or (ii) the tetramer, dimer, and 601 DNA were mixed together at a 1:2.2:1 molar ratio. Both mixtures were then desalted using a linear gradient from 2 M to 150 mM KCl over 36–48 h, followed by dialysis against 0.5× TE. In our hands, refolding octamer together (via method (i)) results in a mixture of hexasome and nucleosome after the salt dialysis reconstitution while refolding tetramer and dimer separately (via method (ii)) results in finer control of the final sample. Samples were then purified with a 10–40% sucrose gradient, which separates residual free 601 DNA and hexasome formed from method (i).

Hexasome samples were made either by isolating hexasome from nucleosome reconstitutions carried out via method (i) or by following method (ii), except mixing tetramer, dimer, and 601 DNA at a molar ratio of 1:1.1:1. Similarly, tetrasome samples were made by following method (ii), except mixing tetramer and 601 DNA at a molar ratio of 1:1 in the absence of dimer. All reconstitutions were purified via sucrose gradient (BioComp Gradient Station, New Brunswick, Canada) (Supplementary Figure S1). Although the DNA footprint of nucleosome, hexasome, and tetrasome are different, all three were prepared using the 147bp Widom 601 sequence in order to hold the total DNA content of the three species constant. It is also important to note that Levendosky *et al.* showed that hexasomes reconstituted using the Widom 601 sequence form a homogeneous population of oriented hexasomes, with the single H2A/H2B dimer preferentially assembling at the TA-rich side of the DNA (15).

Native- and SDS-PAGE were used to assess the formation of nucleosome, hexasome, and tetrasome along with their histone compositions. Bands were visualized with ethidium bromide or Coomassie for native and denaturing gels, respectively. Gels were imaged using an ImageQuant LAS 4000 imager (GE Healthcare). With native-PAGE, the nucleosome runs as the most compact particle, followed closely by hexasome and then tetrasome (Supplementary Figure S1). This supports the model wherein first one and then both arms of DNA open up upon the loss of one or two dimers, respectively, and these changes would lead to more extended structures. Additionally, the nucleosome runs as the densest species on a sucrose gradient, again followed closely by hexasome and then tetrasome (Supplementary Figure S1), which is again consistent with the structural models (1,22). Notably, the tetrasome runs as a collection of bands on native-PAGE, with one major species. The basis of this is unknown, but could be due to differential positioning of the tetramer along the DNA and/or due to the presence of multiple tetramers on a single 147 bp. Tetrasome was observed to be unstable in the presence of KCl, leading to the appearance of free 601 DNA via native-PAGE. Thus, tetrasome samples were only studied in buffers without salt added. SDS-PAGE confirmed the composition of the four histones within the final samples used for experiments (Supplementary Figure S1). The band density was used as a measure of intensity and was quantified using the ImageJ program (NIH). As in (15), H2A and H2B were integrated together due to their lack of resolution. The intensities of gel bands were normalized to H3 to provide a relative intensity, and the average and standard deviation are taken from four gel replicates. Similar to that seen by Levendosky *et al.* (15), when the intensities of the gel bands are normalized to that of H3, the nucleosome contains nearly twice as much H2A and H2B as hexasome (Supplementary Figure S1).

Nucleosome concentrations were determined via UV–vis spectroscopy using the absorbance from the 601 DNA (calculated ϵ_260_ = 2 312 300.9 M^−1^ cm^−1^). Samples were diluted into 2 M KCl prior to concentration measurements in order to promote nucleosome disassembly for more accurate concentration determination.

### NMR spectroscopy data collection and analysis

To obtain backbone assignments for H3 within the context of subnucleosomes, HNCACB and CBCAcoNH spectra were collected on a 360 μM ^13^C/^15^N-H3 hexasome sample (i.e. 720 μM of H3) and a 130 μM ^13^C/^15^N-H3 tetrasome sample (i.e. 260 μM of H3) at 45°C and 37°C, respectively, using a Bruker Avance NEO 600MHz spectrometer. The HNCACB was collected with 32 scans and 88 and 68 total points in the ^13^C- and ^15^N-dimensions, respectively. The CBCAcoNH was collected with 24 (hexasome) or 32 (tetrasome) scans and 88 and 90 total points in the ^13^C- and ^15^N-dimensions, respectively. Assignments at 45°C on ^13^C/^15^N-H3 nucleosome were used from (35). Temperature titration was used to transfer assignments to 25°C and 37°C. Data were processed in NMRPipe (45) and assigned using CcpNMR Analysis (46). Assignments are summarized in Supplementary Figure S5 and Supplementary Table S1. Due to the repetitive and unstructured nature of the H3 tail, there is chemical shift degeneracy in some of the resonances. Associated assignment uncertainty is noted in Supplementary Figure S5 and Supplementary Table S1. Similar to (35), ^1^H/^15^N-HSQC spectra collected on H3K_C_4me3-hexasome were used to help confirm assignments of residues 3–9. Notably, the majority of peaks had degeneracy in C_α_ and C_β_ chemical shifts and thus could not be definitively assigned to one of the two copies of H3 within the hexasome. As noted in the results, these were categorized into subsets referred to as hex-N and hex-T according to amide chemical shift overlap with the nucleosome and tetrasome species, respectively. This is also noted in Supplementary Figure S5 and Supplementary Table S1.

^1^H–^15^N HSQC spectra were collected on ^15^N-H3 nucleosome, hexasome, and tetrasome samples. Samples were exchanged into 20 mM MOPS pH 7, 1 mM DTT, and 1 mM EDTA (with 7 mM NaOH to pH and with some samples also containing 150 mM KCl where noted), and 7% D_2_O was added prior to data collection. The majority of data were collected on a Bruker Avance II 800 MHz spectrometer with cryogenic probe. The spectra of 601 DNA-bound H3(1–44) were collected on a Bruker Avance Neo 800 MHz spectrometer with cryogenic probe. To account for differences between instruments, referencing of an apo-spectrum of ^15^N-H3(1–44) was shifted until spectra overlaid between instruments, and referencing of the 601 DNA-bound spectrum was shifted by the same amount. All NMR data were processed in NMRPipe (45) and analyzed using CcpNMR Analysis (46). The chemical shift difference (Δ*δ*) between samples was calculated by:

$$\begin{equation*}\Delta \delta \; = \sqrt {{{\left( {\Delta {\delta _{\rm H}}} \right)}^2} + {{\left( {0.154\Delta {\delta _{\rm N}}} \right)}^2}} \;\end{equation*}$$

where Δ*δ*_H_ and Δ*δ*_N_ are the differences in the ^1^H and ^15^N chemical shift, respectively, between samples. Data plots were made in Igor Pro (Wavemetrics).

### 15N relaxation experiments

{^1^H}–^15^N steady-state heteronuclear nuclear Overhauser effect (hetNOE) and longitudinal (R_1_) and transverse (R_2_) ^15^N relaxation rates were measured on ^15^N-H3 nucleosome (107 μM) and tetrasome (115 μM) samples in 20 mM MOPS pH7 (with 7 mM NaOH to pH), 1 mM EDTA, 1 mM DTT, 7% D_2_O. Data were collected using standard interleaved Bruker experiments (hsqcnoef3gpsi, hsqct1etf3gpsi3d and hsqct2etf3gpsi3d) at 37°C on a Bruker Avance Neo 800MHz spectrometer with cryogenic probe. HetNOE experiments were collected with an interscan delay of 5 s and 2048 (^1^H) × 512 (^15^N) total points, with acquisition times of 61.4 ms (^1^H) and 95.7 ms (^15^N) and spectral widths of 20.8 ppm (^1^H) and 33.0ppm (^15^N). ^15^N R_1_ experiments were collected with total relaxation loop lengths of 10 (×2), 100 (×2), 200 (×2), 500 (×2), 1000 (×2) and 2000 (×2) ms and an interscan delay of 1 s. ^15^N R_2_ experiments were collected with total relaxation CPMG loop lengths of 16.96 (×2), 50.88 (×2), 67.84 (×2), 101.76 (×2), 152.64 (×2), 203.52 (×2) ms and an interscan delay of 1 s. ^15^N R_1_ and R_2_ experiments were collected with 2048 (^1^H) × 400 (^15^N) total points, with acquisition times of 61.4 ms (^1^H) and 74.8 ms (^15^N) and spectral widths of 20.8 ppm (^1^H) and 33.0 ppm (^15^N).

Spectra were processed with NMRPipe (45) by doubling the size by zero-filling twice (rounding to the nearest power of 2) and using a cosine squared bell window function in both ^1^H and ^15^N dimensions. HetNOE values were calculated from peaks heights, and errors were calculated using standard error propagation within CcpNMR Analysis. Relaxation times were determined from fitting peak heights to a single-exponential decay (without offset) with errors determined via the covariance method using CcpNMR Analysis (46). The T_1_/T_2_ ratio was calculated from these fit values, and error was propagated from individual T_1_ and T_2_ fits using standard error propagation. Relaxation rates were calculated as the inverse of relaxation times, and error was propagated from T_1_ and T_2_ using standard error propagation. Residues with peak overlap are denoted in Figure 3. These residues were analyzed in the same manner as the rest, but relaxation rates are likely influenced from convolution with neighboring peak. Residues are included in the overall analysis with that caveat. The two peaks of K36 in ^15^N-H3 nucleosome were analyzed separately and are plotted at positions 36 and 36.5 in Figure 3.

### Trypsin proteolysis assays

Trypsin proteolysis was used as a probe for site exposure on histone tails within nucleosomes and subnucleosomes. Digests were carried out on samples of reconstituted nucleosome, hexasome, and tetrasome at a fixed concentration of 3 μM in 20 mM MOPS pH 7, 1 mM EDTA and 1 mM DTT.

Assays conducted at multiple ratios of trypsin were carried out at room temperature in 10 μl reactions with 30, 6 and 1.2 nM trypsin (Pierce product 90057, MS grade). Gel samples were taken prior to addition of trypsin (taken as *t* = 0) and 20 min after mixing with trypsin, when they were immediately mixed with 5× SDS loading dye and heated to 95°C for 10 min. Gel samples contained 13 pmol of the particular nucleosome species and were run on 18% tris-glycine SDS-PAGE gels followed by Coomassie staining. To check stability of the species over the course of the assay, 1.3 pmol of the particular nucleosome species from before and after the assay were run on 5% native-PAGE gels and visualized with ethidium bromide.

Experiments with full time courses were conducted at 6 nM trypsin (1:1/500 molar ratio of nucleosomal species:trypsin) in 80 μl reactions. Samples were incubated in a thermomixer (Eppendorf) at 25°C while shaking at 350 rpm. Gel samples were taken prior to addition of trypsin (taken as *t* = 0) and at *t* = 2, 5, 10, 15, 20, 30 and 50 min after mixing with trypsin. Samples were quenched by immediately mixing with 5× SDS loading dye and heating to 95°C for 10 min. Gel samples contained 15 pmol of nucleosome or subnucleosome and were run on 18% tris-glycine SDS-PAGE gels and visualized with Coomassie stain. To check stability of the species over the course of the assay, 1.5 pmol of nucleosome or subnucleosome from before and after the assay were run on 5% native-PAGE gels as before.

All experiments were run in triplicate. The native-PAGE confirmed that the nucleosomes, hexasomes and tetrasomes remained largely intact over the course of the experiments.

### Analysis of trypsin proteolysis assays

#### Gel imaging

Gels were imaged using an ImageQuant LAS 4000 imager (GE Healthcare). The band density of full-length H3 was used as a measure of intensity and was quantified using the ImageJ program (NIH). The fraction of full-length H3 remaining at a given time was taken as the ratio of the band densities of full-length H3 at that time point and prior to the addition of trypsin.

#### Digests at multiple concentrations of trypsin

The amounts of full-length H3 remaining after 20 min digestion at the three concentrations of trypsin were compared. To determine whether the extent of digestion was significantly different between the nucleosome and subnucleosomes at each concentration of trypsin, a two-way ANOVA followed by a tukey post-hoc analysis was run using R on the data sets that were collected in triplicate. A cutoff of *P* < 0.05 was used for significance.

#### Proteolysis kinetics

We treated the experimental data for site exposure on the H3 tails probed via trypsin proteolysis in the same manner as site exposure on DNA probed via restriction enzymes (47,48) and in a similar manner as site exposure on histone tails probed via chemical modification (30,37). These other experiments were designed for digestion and modification at single sites within the nucleosome. Although trypsin has many target sites within the histones, only the general proteolysis of the H3 tail is monitored by following the amount of full-length H3 remaining in the sample at a given time. The subsequent analysis makes several assumptions. First, we make the assumption that the system is in the limit of rapid conformational pre-equilibrium. In this limit, there is a first-order dependence of the observed rate constant (*k*_obs_) on enzyme concentration. Site exposure on nucleosomal DNA and H2B tails were shown to be in limit of rapid pre-equilibrium within the experimental contexts of (48) and (37), and the assumption of rapid pre-equilibrium was made for the H3 tail in (30). Thus, it is likely that site exposure on nucleosomal and subnucleosomal H3 tails is also in the limit of rapid pre-equilibrium in the proteolysis experiments described here. Although full kinetic data sets were only collected at a single concentration of trypsin, the single timepoint data collected at three trypsin concentrations suggests a linear relationship between k_obs_ (where the natural log of the fraction of full length H3 remaining is taken as a very rough proxy for *k*_obs_) and enzyme concentration (Supplementary Figure S11). An additional assumption is that the concentration of exposed histone tails is much less than the *K*_m_ of trypsin such that the free concentration of enzyme is equivalent to the total concentration of trypsin in the sample. Additional assumptions are that the concentrations of exposed H3 tail and H3 tail-trypsin complex are at steady state. Lastly, the assumption was made that the proteolysis events report predominantly on site exposure within the native conformation of the H3 tail within nucleosome or subnucleosome rather than site exposure that has been altered by a preceding cleavage event.

The average and standard deviation of the fraction of full-length H3 remaining at each time point was calculated from the triplicate data sets. The *k*_obs_ were determined from a weighted single exponential fit of the data average. The fit was additionally constrained to decay to zero and to have *y*-intercept ≤1. The *y*-intercept was allowed to be less than one to account for the possibility that the initial mixing of the sample led to dissociation of a subpopulation of particles. Under these constraints, the tetrasome experiment fit with a *y*-intercept of 1.0 ± 0.2 and the nucleosome experiment fit with a *y*-intercept of 0.87 ± 0.04. In studies of DNA site exposure, up to 10% of nucleosomes were observed to dissociate due to rapid mixing (49).

The ratio of site exposure equilibrium constants for tetrasome and nucleosome was taken as the ratio of the *k*_obs_ fit from the data sets for tetrasome and nucleosome. This only holds if the assumptions detailed above are valid. The error in the ratio of site exposure equilibrium constants was propagated from the error in the fits for the *k*_obs_ from the two data sets.

### Preparation/generation of canonical and subnucleosomal particles for molecular dynamics simulations

Nucleosome models were constructed by taking a Widom 601 DNA molecule from PDB 3MVD and aligning the DNA onto the histone core coordinates from the 1KX5 PDB (50,51). Extended states of the H3 tails were built using MODELLER (52). Hexasome models were generated by removal of the H2A/H2B dimer from the nucleosome TA-poor side, followed by implicit solvent molecular dynamics (MD) runs to create more open DNA structures (22). These initially involved imposing position restraints on the first 107 bp of DNA while allowing the remaining 40 bp of DNA to relax for two ns in an implicit solvent environment with Watson–Crick base pair restraints. This DNA geometry was then aligned with the histone hexamer to generate a crude hexasome intermediate, which was then simulated for 20 ns in an implicit environment to obtain a relaxed state with an extended DNA arm. Similarly, the tetrasome was generated by keeping only the H3/H4 tetramer and allowing 40 bp of DNA from both sides of the nucleosome to relax during simulations. The starting tetrasome conformation had only ∼66 bp of DNA wrapped around the H3/H4 tetramer, in accordance with the experimentally probed tetrasomal geometry (22). The initial conformations of the canonical and subnucleosomal particles are given in Supplementary Figure S8.

### Simulation methods

All simulations were conducted in the CUDA-enable PMEMD engine of the AMBER software suite (v18) (53,54). The Amber 14SB and BSC1 forcefields parameters were used for the protein and DNA respectively (55,56). Implicit simulations were performed using mbondi3 and igb = 8 (57). For explicit solvent simulations, all systems were neutralized and solvated with TIP3P waters and a 0.15 M KCl environment (58,59). A 4-fs time-step was used in conjunction with SHAKE and hydrogen mass repartitioning for all simulations (60,61). The use of hydrogen mass repartitioning has been shown to provide good agreement with conventional simulations, while allowing for nearly twice the simulation speed (60). All systems were energy minimized for 5000 steps with a solute harmonic restraint of 10 kcal/mol/Å^2^, followed by 5000 steps with no restraints. For equilibration, we first performed 100 ps of constant volume simulations while the temperature was gradually heated from 10 to 300 K. Then, the heavy atom restraints were gradually released over 500 ps of NPT run. In explicit solvent simulations, pressure was controlled via a monte carlo barostat with a target pressure of 1 atm and a relaxation time of 3.0 ps^−1^. Production runs were performed at 300K using a Langevin thermostat (62). We performed ten, 250 ns simulations per system in the NPT ensemble, accumulating 7.5 μs of sampling across all the three systems. Although simulations were initiated from the same initial configurations, we noted in our previous study that the use of random initial velocities provide roughly the same amount of conformational heterogeneity in the H3 tails as starting simulations from different extended states (35). Trajectories were recorded every 10 ps and visualized using VMD (63) and PyMol (64). Analysis was performed on the last 150 ns of the simulations, allowing for 100 ns of equilibration.

### Simulation analyses

Convergence of molecular dynamics simulations trajectories was monitored via rms average correlation (RAC) analysis, which is implemented in CPPTRAJ (65,66). This is a pseudo-autocorrelation function for root mean square deviation (RMSD) values and measures the overall average structure at different time intervals within a single trajectory. The RAC is calculated according to:

$$\begin{equation*}{\rm RAC}(\tau ) = \frac{{\sum\nolimits_{t = 0}^N {{\rm RMSD}({\rm AvgCrd}(t,t + \tau ))} }}{{N - \tau + 1}}\end{equation*}$$

where ($\tau$) is a time interval and a straight running coordinate averaging is performed using this time interval over the entire trajectory by fitting to the averaged structure obtained from the individual trajectory. For example, at ($\tau \;$= 1), RAC is a standard average RMSD over the entire trajectory. The decay of RAC values for all the independent simulations conducted in this study is presented in supporting information (Supplementary Figure S2). The last 150 ns belongs to the equilibrated portion of the trajectory. Further, solvent accessibility of H3 tails and radius of gyration were calculated as a function of time to observe the collapse of H3 tails and stability of the simulations (Supplementary Figures S3 and S4).

Root mean square fluctuation (RMSF) analysis was performed on the H3 proteins, the first 37 residues defined as the tails, and residues beyond this defined as the core region. For RMSF calculations, trajectories were aligned to the H3 and H4 globular domain. Translations and rotations were removed by least squares fitting the backbone of the H3 and H4 histone, and RMSFs were computed on the Cα atoms. Reported RMSFs are the average of all 10 simulations. Errors are presented as the standard error of the mean obtained from 10 samples for each tail. Kullback-Leibler Divergence was performed using internal coordinates to compare conformational ensemble across the systems (67). Contact analysis between H3 tails and DNA was performed using MDanalysis (68) and in-house python scripts, where contacts were defined as between heavy atoms of H3 tail residues that were within a distance of 4.5 Å from DNA heavy atoms. Interaction energies between the H3 tails and DNA were determined from the sum of tail residue contributions to DNA binding via an MM-GBSA (Molecular Mechanics Generalized Born Surface Area) analysis with igb = 5 and a salt concentration of 0.15 M (69). Error bars represent the standard error of the mean, with a decorrelation time of 10 ns that is based on a statistical inefficiency test of MM/GBSA values.

### Principal component analysis (PCA)

PCA is often used to extract slow and functionally important motions of biomolecules from the MD trajectories by dimensionality reduction techniques. First, the covariance matrix of atomic positions of Cα atoms of H3 tails (residues 1–37) was built, and the eigenvectors of this matrix (also known as principal components) describes the concerted motion of the system. In this study, PCA was performed using GROMACS-2016.3 to investigate the similarities and differences in the conformational ensembles of H3 tails across the systems. Here, PCA was performed with respect to the H3 Cα tail atoms.

## RESULTS

### The H3 tail conformation is sensitive to nucleosome composition

**Figure 1.**
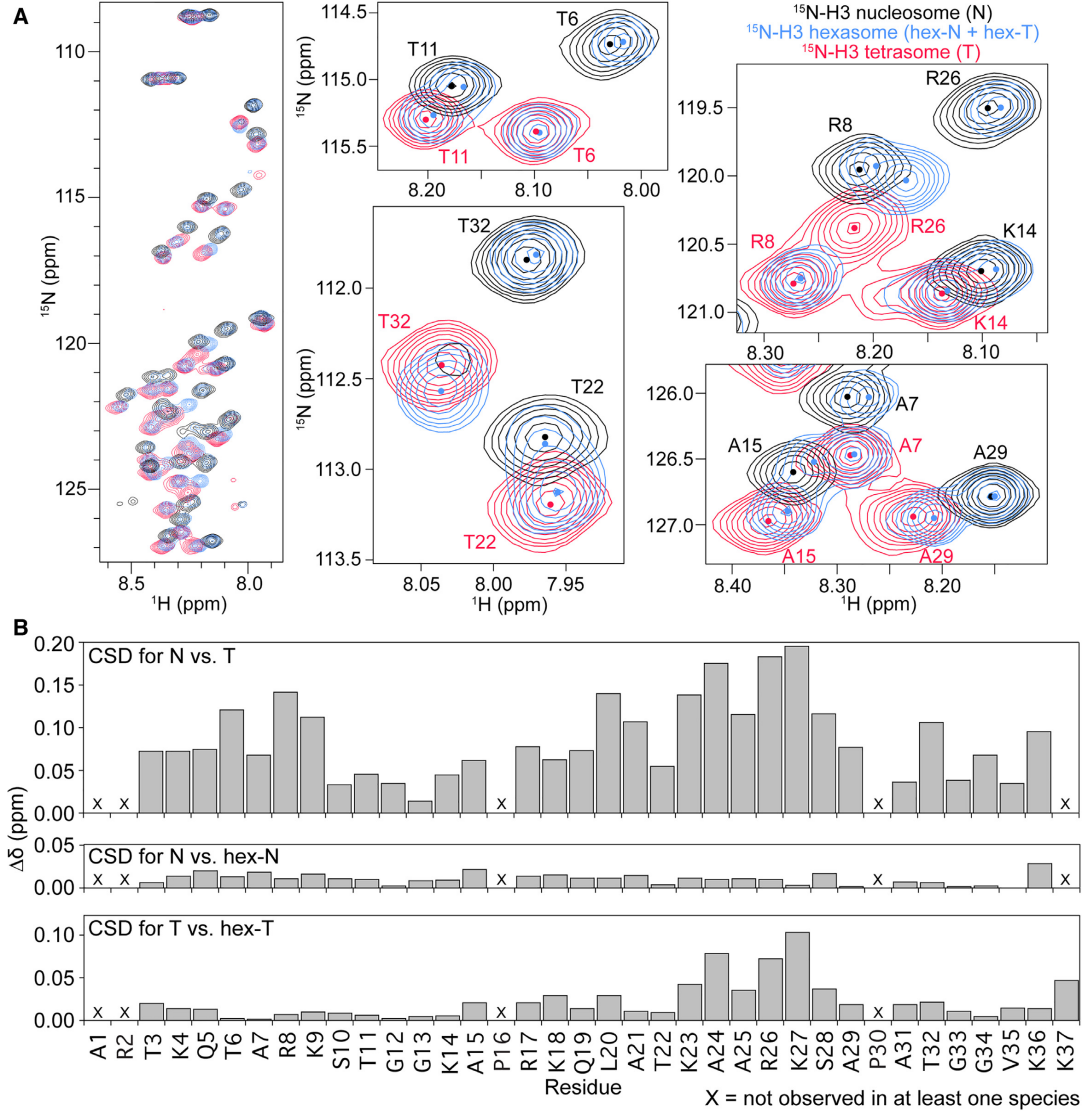
H3 tail conformation is distinct between nucleosomal species. (**A**) Overlay of ^1^H/^15^N-HSQC spectra collected on ^15^N-H3-labeled versions of the three nucleosomal species, nucleosome (black), hexasome (blue) and tetrasome (red). Comparison of the spectra indicates that the H3 tail exists in different conformational ensembles between the nucleosome and tetrasome and suggests that hexasome contains one copy of H3 in a similar conformational ensemble as nucleosome and one copy of H3 in a similar conformational ensemble as tetrasome. Expanded regions of the overlay are shown for selected residues for closer comparison of histone tail states. Small circles mark the approximate center of each peak to aid in the spectral comparison. These spectra were collected on 44 μM ^15^N-H3 nucleosomal species in 20 mM MOPS pH 7, 1 mM EDTA, 1 mM DTT, 7% D_2_O at 37°C and on an 800 MHz spectrometer. (**B**) Chemical shift differences (Δδ) between the nucleosome and tetrasome H3 tails (top), the nucleosome and hex-N H3 tails (center), and the tetrasome and hex-T H3 tails (bottom). This plot is shown as a function of H3 tail residue.

To compare H3 tail conformational states between the canonical nucleosome core particle and the sub-nucleosome species hexasome and tetrasome, we used NMR spectroscopy. Nucleosome, hexasome, and tetrasome were reconstituted using H3/H4 tetramer containing ^15^N-labeled H3, and varied amounts of H2A/H2B dimer as required to obtain a given species (Supplementary Figure S1). All species were reconstituted with the 147 bp Widom 601 DNA (see Materials and Methods section for details). Initial comparison of the ^1^H,^15^N-heteronuclear single quantum coherence (HSQC) spectra of each species (Figure 1 and Supplementary Figure S5) reveals unique spectral attributes for the H3 tails within each species, indicating that nucleosome composition alters the conformation of the H3 tails.

One major difference between the spectra is in the number of unique peaks observed: 32 peaks for ^15^N-H3 nucleosome, 33 peaks for ^15^N-H3 tetrasome and 65 peaks for ^15^N-H3 hexasome (Figure 1 and Supplementary Figure S5). To better understand these differences, we carried out backbone assignments of the resonances. We previously assigned the ^15^N-H3 nucleosome peaks to H3 tail residues, with only a single set of peaks observed for the two tails (35). For ^15^N-H3 tetrasome, a single set of peaks was also observed for residues spanning 3–36, and an additional peak was observed corresponding to Lys37 (Supplementary Figure S5). The single set of peaks for both ^15^N-H3 nucleosome and ^15^N-H3 tetrasome indicates that within each of these nucleosomal species the two H3 tails experience largely the same chemical environment (Figure 1A, black and red spectra), which is consistent with the structural pseudo-symmetry within each of these species. Notably, a subset of peaks within ^15^N-H3 nucleosome (residues Q5, A25, R26, S28 and K36) are broadened approaching a doublet. This suggests two distinct but very similar conformational ensembles, especially near the core. This is only observed for T3 within ^15^N-H3 tetrasome. In addition, minor peaks are observed in both the ^15^N-H3 nucleosome and ^15^N-H3 tetrasome. However, these minor peaks could not be definitively assigned and thus their identity remains unknown.

Assignments for ^15^N-H3 hexasome show that the 65 observed peaks all correspond to the H3 tails, but in contrast to the nucleosome and tetrasome, two highly distinct peaks are observed for most residues in the H3 tail (Figure 1A and Supplementary Figure S5, blue spectrum). This indicates two distinct states (or ensembles of states) of the tails within the hexasome. The multiple peaks could be explained by (i) the two H3 tails experiencing distinct chemical environments or ii) interconversion of both of the H3 tails between two states that is slow on the NMR timescale. Importantly, Levendosky *et al.* elegantly showed that hexasomes reconstituted using the Widom 601 sequence preferentially assemble with the single H2A/H2B dimer at the TA-rich side of the DNA (15). In addition, it has been shown that the 601 DNA asymmetrically unwraps from the histone core, becoming more accessible on the side of the particle lacking the H2A/H2B dimer (15,27,70). Thus, we hypothesize that the two sets of peaks arise from each of the H3 tails adopting a distinct conformational ensemble, dependent on the presence or absence of the adjacent H2A/H2B dimer.

Additional insight into the conformations of the H3 tails within the different nucleosomal species can be gained by comparing chemical shifts of H3 tail resonances between nucleosome, hexasome, and tetrasome (Figure 1A and Supplementary Figure S5). Overlay of spectra for the nucleosome and tetrasome reveals that, even though the number of peaks is the same, there are substantial differences in the chemical shift of all residues (Figure 1A, compare black and red spectra). This reveals that loss of both H2A/H2B dimers leads to a change in the chemical environment of the H3 tails. Overlay of the hexasome spectrum reveals something quite striking: in the spectrum for ^15^N-H3 hexasome, half of the peaks overlay well with the nucleosome spectrum and the other half overlay well with the tetrasome spectrum (Figure 1A). Furthermore, these two sets of peaks correspond to residues of a full H3 tail (i.e. correspond to residues H3 1–36 or 37). Together, this leads us to hypothesize that one H3 tail in the hexasome adopts a conformation similar to the nucleosome and the other adopts a conformation similar to the tetrasome. As such, these will be referred to as the hex-N and hex-T tails, respectively.

To better quantitate these comparisons, chemical shift differences (CSDs or Δδs) between the nucleosome and tetrasome peaks, the hex-N and nucleosome peaks, and the hex-T and tetrasome peaks were calculated (Figure 1B and Supplementary Figure S6). Peaks for the nucleosome versus tetrasome had an average Δδ = 0.09. The majority of resonances had Δδ>0.05, with the largest differences observed for residues K23-S28, indicating substantial differences in conformation. In contrast, peaks for the hex-N tail as compared to the nucleosome have Δδ < 0.03 along the entire length of the tail, indicating a highly similar conformation. While compared to the tetrasome, the majority of the hex-T tail peaks also have Δδ < 0.03, there are some residues that exhibit greater differences from the tetrasome tail. In particular, residues K23-S28 and K37 have Δδ > 0.03. These differences may reflect known differences in overall stability, positioning and dynamics of the tetrasome (1,6,22,70). In addition, the chemical shift differences between the hex-N and tetrasome peaks, and the hex-T and nucleosome peaks show similar differences to those observed between nucleosome and tetrasome (average Δδ = 0.09 and 0.07, respectively, Supplementary Figure S6). This further supports the conclusion that one H3 tail adopts a nucleosomal-like state and the other adopts a tetrasomal-like state.

Altogether, these results strongly suggest that while the H3 tails adopt distinct conformational ensembles between nucleosome and tetrasome, the two tails are largely symmetric in both. In contrast, hexasome H3 tails are conformationally asymmetric, with one H3 tail adopting a nucleosome-like ensemble (hex-N) and one H3 tail adopting a tetrasome-like (hex-T) ensemble.

### Loss of the H2A/H2B dimer increases the dynamics of the H3 tail

**Figure 2.**
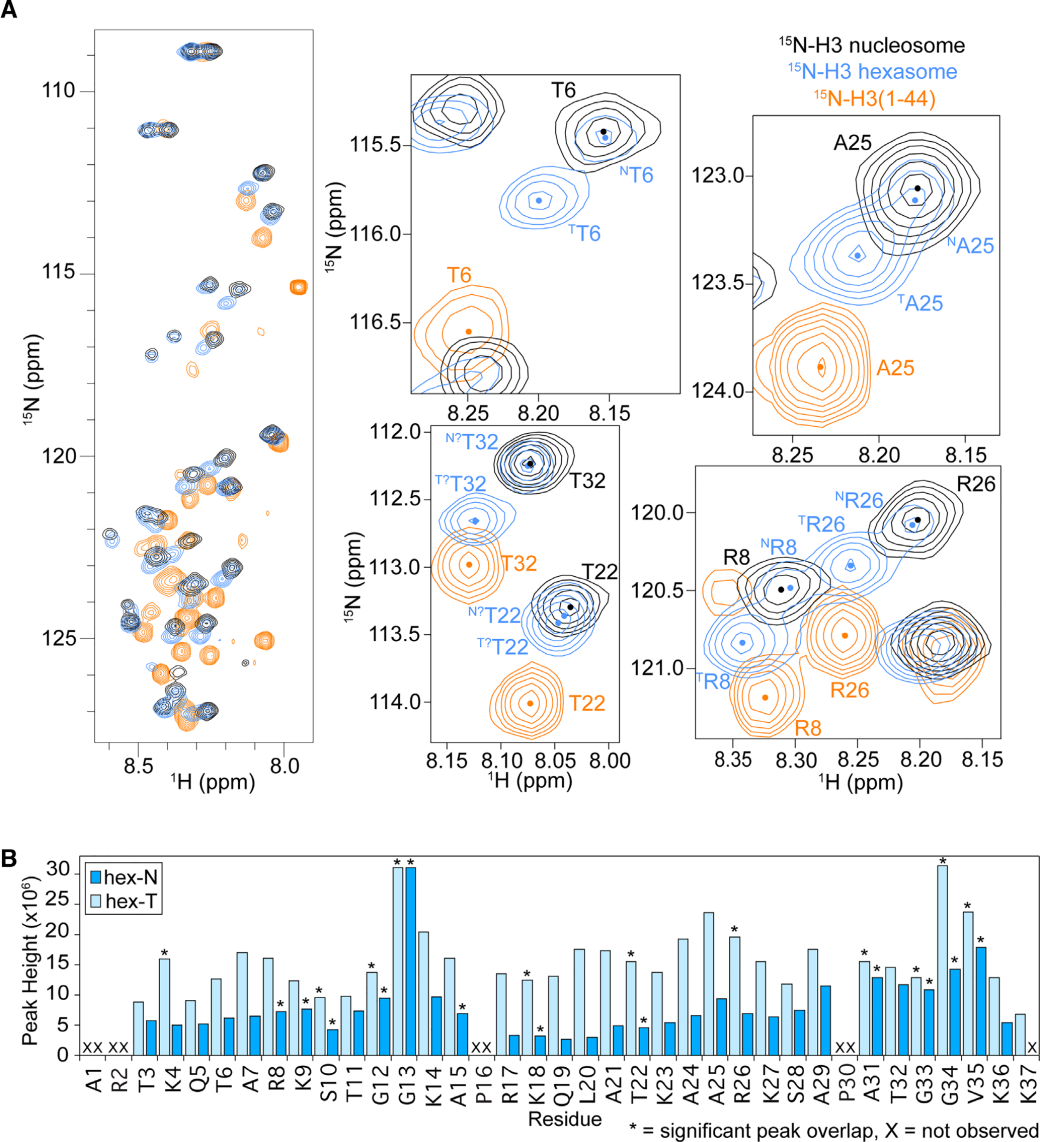
Tetrasomal tail is more dynamic. **(A)** H3 tail conformation within tetrasome more closely mirrors H3 tail peptide. Overlay of ^1^H/^15^N-HSQC/HMQC spectra collected on ^15^N-H3 nucleosome (black), ^15^N-H3 hexasome (blue), and ^15^N-H3(1–44) (orange). Comparison of the spectra shows that, in general, the tetrasomal H3 tail experiences a more similar chemical environment to the H3 tail peptide than does the nucleosomal H3 tail, which suggests a more extended tail ensemble within the tetrasome than the nucleosome. Expanded regions of the overlay are shown for selected residues for closer comparison of histone tail states. Small circles mark the approximate center of each peak to aid in the spectral comparison. These spectra were collected on 44μM ^15^N-H3 nucleosome species or 110 μM ^15^N-H3(1–44) in 20 mM MOPS pH 7, 150 mM KCl, 1 mM EDTA, 1 mM DTT, 7% D_2_O at 25°C and on an 800 MHz spectrometer. (**B**) Peak intensity (height) is plotted as a function of residue for the hex-N (blue) and hex-T (light blue) H3 tails within the hexasome for the spectrum shown in Figure 1A (blue). Residues that are not observed in the spectra are marked with an ‘X’. Residues with significant overlap that prevents accurate determination of peak height are marked by ‘*’.

Analysis of NMR spectra of each species also provides insight into the dynamics of the H3 tails. Notably, clear signal for K37 is observable in spectra of ^15^N-H3 tetrasome as compared to nucleosome. The appearance of Lys37 indicates that the tail is more dynamic near the particle core in the tetrasome relative to the nucleosome. Consistent with one tail being in a tetrasomal-like state in the hexasome, only a single peak is observed for Lys37 in the ^15^N-H3 hexasome (Supplementary Figure S5). Comparing amide chemical shifts between the hexasome and a peptide corresponding to the H3 tail (residues 1–44) (Figure 2A) reveals that, in general, the hex-T H3 peaks lie along a near-linear trajectory between the nucleosome (or hex-N) and peptide peaks, though not fully reaching the peptide chemical shifts. This suggests that, upon loss of the H2A/H2B dimer, the conformational equilibrium of the H3 tail is shifted towards a more conformationally unrestricted state. Notably, the hex-T chemical shifts are highly similar to chemical shifts for the H3 tail peptide bound *in-trans* to DNA or *in-trans* to a tailless nucleosome (Supplementary Figure S7), suggesting that though more conformationally unrestricted they are in a DNA-bound state. Additional insight into the dynamics of the tails can be garnered from comparison of peak intensity. Peak intensity reports on intrinsic dynamics but is also influenced by overall tumbling. Thus, we focused on the two sets of peaks in the hexasome since they have the same overall tumbling. Analysis of the intensities of the hexasome peaks reveals that the hex-T subset of peaks is on average 2.4-fold more intense than the hex-N subset of peaks. This difference is the largest (on average 2.6-fold) for peaks corresponding to the first 29 residues of the H3 tails (Figure 2B). This suggests that the hypothesized tetrasomal H3 tail is more conformationally dynamic than the hypothesized nucleosomal H3 tail.

**Figure 3.**
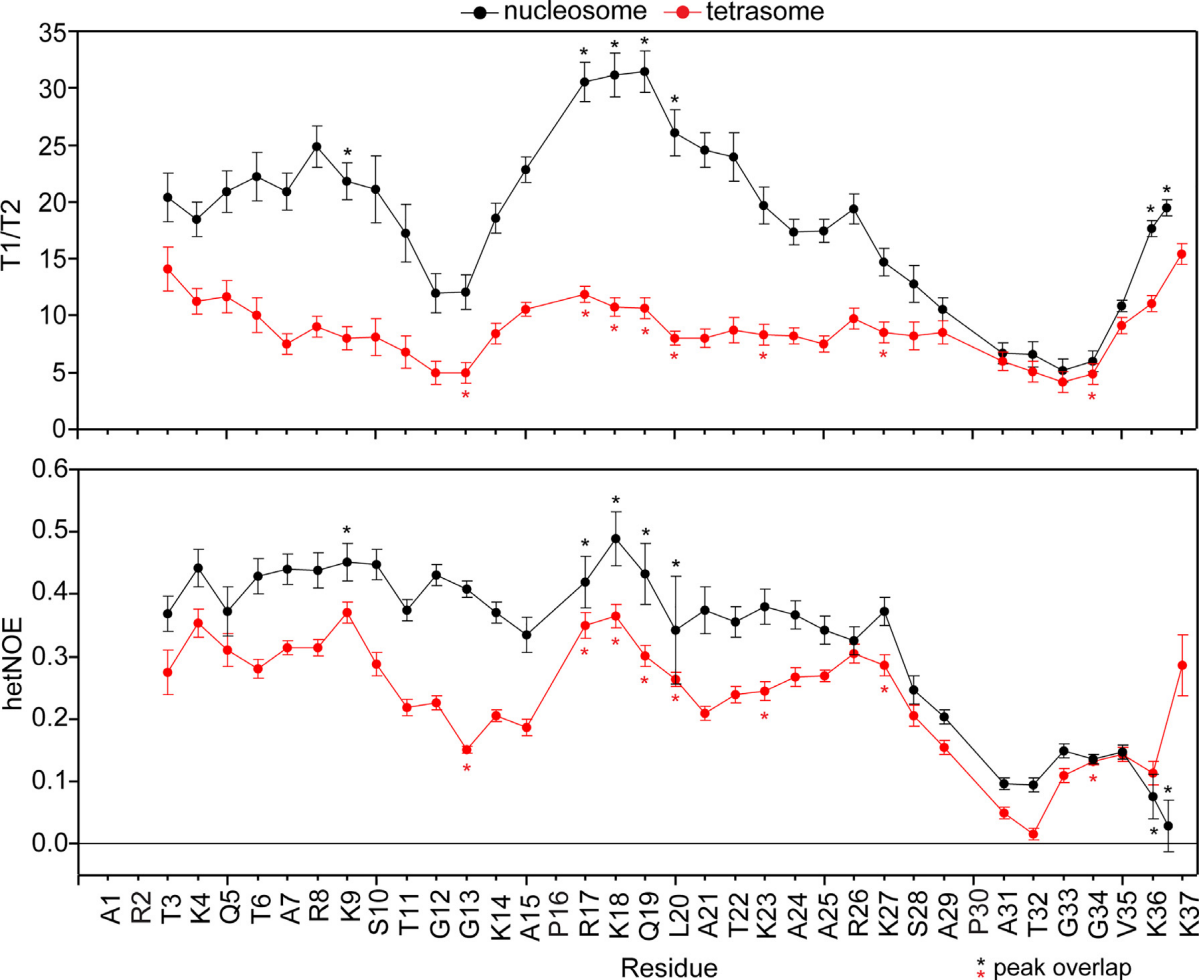
H3 tail picosecond-nanosecond timescale dynamics are distinct between nucleosome and tetrasome. {^1^H}–^15^N hetNOE values and ratio of ^15^N relaxation times (T_1_/T_2_) are plotted as a function of H3 tail residue for ^15^N-H3 nucleosome (black) and tetrasome (red). Residues with peak overlap are marked by ‘*’.

To further quantify this, we measured ^15^N T_1_, ^15^N T_2_ and {^1^H}–^15^N heteronuclear nuclear Overhauser effect (hetNOE) relaxation parameters for the ^15^N-H3 nucleosome and ^15^N-H3 tetrasome, which report on picosecond-nanosecond dynamics (Figure 3, Supplementary Figure S8). The ratio of T_1_/T_2_ is proportional to the rotational correlation time experienced by each residue, which includes contributions from overall tumbling and internal molecular motions. The pattern across the H3 tails is similar between nucleosome and tetrasome. As has been previously reported for the nucleosome (36), the tails are observed to be more restricted at basic segments and less restricted at uncharged segments. At these molecular sizes, the hetNOE values are more sensitive to changes in internal motions than to changes in overall rotational diffusion (71). For both the nucleosomal and tetrasomal H3 tails, all hetNOE values are within the range of 0–0.5. This supports that the H3 tails are not rigid (which would lead to values near 0.8) but are also not conformationally unrestricted (which often leads to values < 0). This is consistent with a model in which the H3 tails dynamically interact with nucleosomal DNA. However, hetNOE values are overall lower for the tetrasomal H3 tails, supporting that they are less restricted than the nucleosomal H3 tail, particularly for residues 3–25. Notably, both species display a dip in the hetNOE profile for residues 31–36, corresponding to an increase in mobility in this region and supporting a flexible ‘hinge’ region for the H3 tail at residues 31–36. This is in agreement with recent papers (34,72) and with cross-linking data which indicate that the H3 tail can either extend toward the dyad or fold back onto the nucleosomal core (43). The smaller overall T_1_/T_2_ values observed for the tetrasome are likely due to a combination of faster overall tumbling (expected for the lower molecular weight particle) and the increase in ps-ns flexibility also reported on by the hetNOE.

Based on these results, we hypothesize that the tetrasomal state of the H3 tail is still bound to DNA, but is more conformationally dynamic on the DNA than the nucleosomal state of the tail on the ps-ns timescale. We further hypothesize that this is due to unwrapping and subsequent lowering of DNA density near the tail, suggesting that the H3 tails sample a conformational ensemble that is linked to the conformation of the DNA.

### Loss of H2A/H2B dimer increases H3 tail conformational fluctuations in MD simulations

To further investigate the conformation and dynamics of the H3 tails in sub-nucleosomes, 10 × 250 ns all-atom molecular dynamics (MD) simulations were carried out on nucleosome, hexasome, and tetrasome (Supplementary Figures S2–S4). We previously observed that the H3 tails in the nucleosome quickly adopt a DNA-bound state no matter their starting conformation. However, multiple DNA-bound states were observed across several simulations with little energetic difference between them. Combined with NMR data, this led us to propose that the H3 tails adopt a fuzzy complex with DNA in the nucleosome context, interacting robustly but adopting a heterogenous and dynamic ensemble of DNA-bound states. In agreement with NMR data, we observe that in all simulations of the hexasome and tetrasome, the H3 tails bind to the DNA within 100 ns similar to what was observed with the nucleosome. Analysis of the end-state of all simulations reveals that in the nucleosome, hexasome, and tetrasome, the H3 tails adopt a heterogeneous ensemble of DNA-bound states (Supplementary Figure S9).

**Figure 4.**
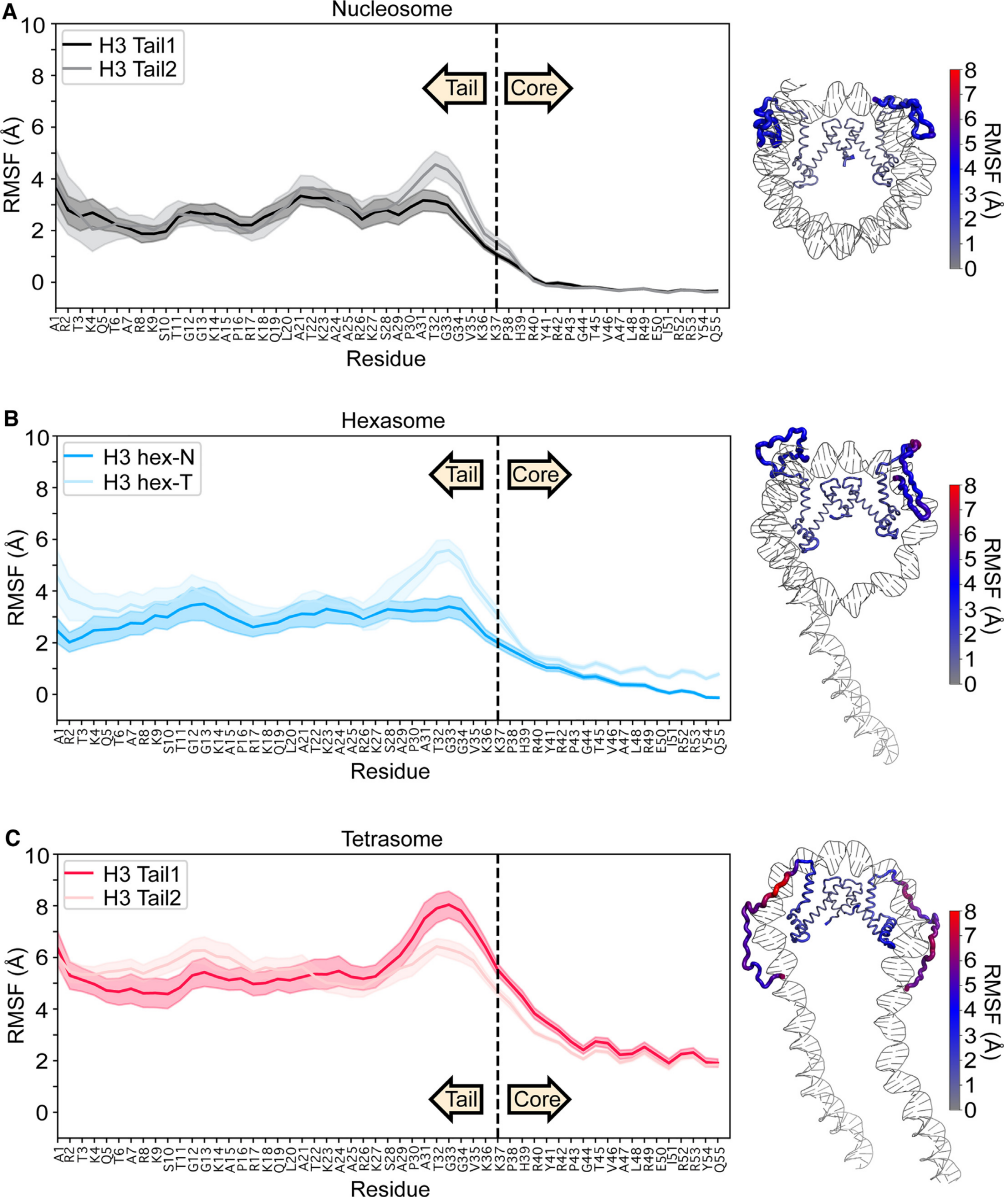
Residue-wise root mean square fluctuation (RMSF) values obtained from the equilibrated portion of MD trajectories. Plots are for (**A**) nucleosome, (**B**) hexasome and (**C**) tetrasome with the average of 10 simulations plotted as a solid line and the standard error of the mean shaded. Data are plotted for the first 55 residues of H3, where residues 1–37 and 38–55 are defined as tail and the initial region of the core, respectively. RMSF values are also plotted on a representative end-state structure of each nucleosomal species. The thickness and color (see key) of the cartoon backbone represents the RMSF value for a given residue, where thicker indicates larger RMSF. H3 is the only histone displayed in the image for ease of visualization.

To assess the conformational dynamics of these DNA-bound states, the average root mean square fluctuation (RMSF) of Cα atoms of each tail over the 10 simulations for each species was calculated. These report on the dynamics of the tails with respect to the histone core. For all tails, average RMSF values were substantially greater than RMSF values for residues in the histone core indicating greater relative conformational dynamics (Figure 4). In the nucleosome, the two H3 tail RMSFs are similar with mean values of 3.0–6.0 Å, indicating a similar degree of conformational dynamics of each tail. In contrast, the initial portion of the H3 core (the α_1_ helix, residues 44–55) has average fluctuations of 0.7 Å. In the tetrasome, both H3 tails also have a similar degree of dynamics relative to each other, but are substantially more dynamic than the nucleosome tails with calculated RMSFs between 5.5 and 10.0 Å. This indicates that removal of the H2A/H2B dimer increases the conformational dynamics of the H3 tails relative to the histone core. Fluctuations in the H3 α_1_ helix were also increased to, on average, 1.8 Å. The calculated RMSF values for the hexasome revealed that, in contrast to the tetrasome and nucleosome, there is a difference between the dynamics of the two tails. The RMSF values (3.0–4.5 Å) for the hex-N H3 tail are similar to values for the nucleosomal H3 tail, with the exception of residues A29-V35 at the end of the tail. In contrast, the hex-T H3 tail shows a significant increase in flexibility (mean values of 3.5–5.5 Å). Similar to the tetrasome, the hex-T H3 α_1_ helix also has a marked increase in flexibility, with RMSF values of 3.0 Å. Altogether this indicates that removal of the H2A/H2B dimer leads to an increase in the H3 α_1_ helix and tail dynamics, and that in the hexasome this introduces asymmetry.

To further ascertain the nature of these increased dynamics, we analyzed the internal motions of the tail residues by comparing the individual dihedral motions across all 10 simulations for each species and quantifying with Kullback–Leibler divergence calculations (67). These calculations are distinct from the previously discussed RMSFs, as they compare differences in local motions, whereas the RMSF is based on cartesian-coordinates and thus computes flexibility of global motions. Results show only a few statistically-significant differences between the nucleosome, hexasome, and tetrasome tails (Supplementary Figure S10). Therefore, while RMSF calculations show that global motions with respect to the core are increased in the hex-T and tetrasomal H3 tails, the differences in localized dihedral motion appear to be relatively minor on the timescale of the simulations. Together, RMSF and KLD analyses suggest that the increased fluctuations observed upon loss of the H2A/H2B dimer are largely due to increased sampling of conformational space relative to the histone core that is linked to increased dynamics of the bound DNA itself ((22) and Supplementary Figure S11)) and do not include a significant contribution from changes in localized dynamics of the DNA-bound states.

### H2A/H2B dimer loss leads to more extended and solvent-exposed states of the H3 tail

To further understand the impact of dimer loss on the H3 tail conformational ensemble, the average inter-residue distances along the H3 tails were calculated, which report on the tail compactness (Supplementary Figure S12). Results show that in all systems, the H3 tails are devoid of any secondary structure elements, consistent with our previous NMR results and simulation studies (35). Compared to the nucleosome, there are decreased long-range intramolecular contacts in the tetrasomal tails, indicating that the H3 tails adopt less-compact conformations (that is, they are more extended) upon loss of the H2A/H2B dimer (Supplementary Figure S12). In the hexasome, the two tails are conformationally asymmetric, with the hex-T tails resembling the tetrasome with fewer long-range intramolecular contacts as compared to the hex-N tails. Interestingly, the hex-N tails adopt even more compact conformations than the nucleosomal tails. Together, this analysis suggests that H2A/H2B dimer loss and DNA opening modulate the conformational ensemble of the adjacent H3 tail towards more extended states along the DNA. While in the hexasome, the H3 tail of the wrapped side becomes more compact. To gain insights into the structures that the bound H3 tails adopt across the system, a principal component analysis (PCA) was performed on the conformations collected from the last 150 ns of the trajectories (Supplementary Figure S13). In this analysis, structures were aligned to the tail residues only, and therefore it only reports on intra-tail motions rather than tail motions relative to the histone core. Principal Component (PC1) and PC2 describe an extension motion and the orientation of H3 tails, respectively. We observe an increase in the negative PC1 values for tetrasomal and hex-T H3 tails, suggesting more extended conformations relative to the hex-N and nucleosomal tails. We note that the hex-N H3 tail adopted states with larger positive PC1 values, which corresponds to more compact states than nucleosomal tails and is in agreement with the inter-residue distance plot of hex-N (Supplementary Figure S12). PC2 corresponds to a twisting motions of the tail's backbone along its major axis, and has similar sampling between species.

**Figure 5.**
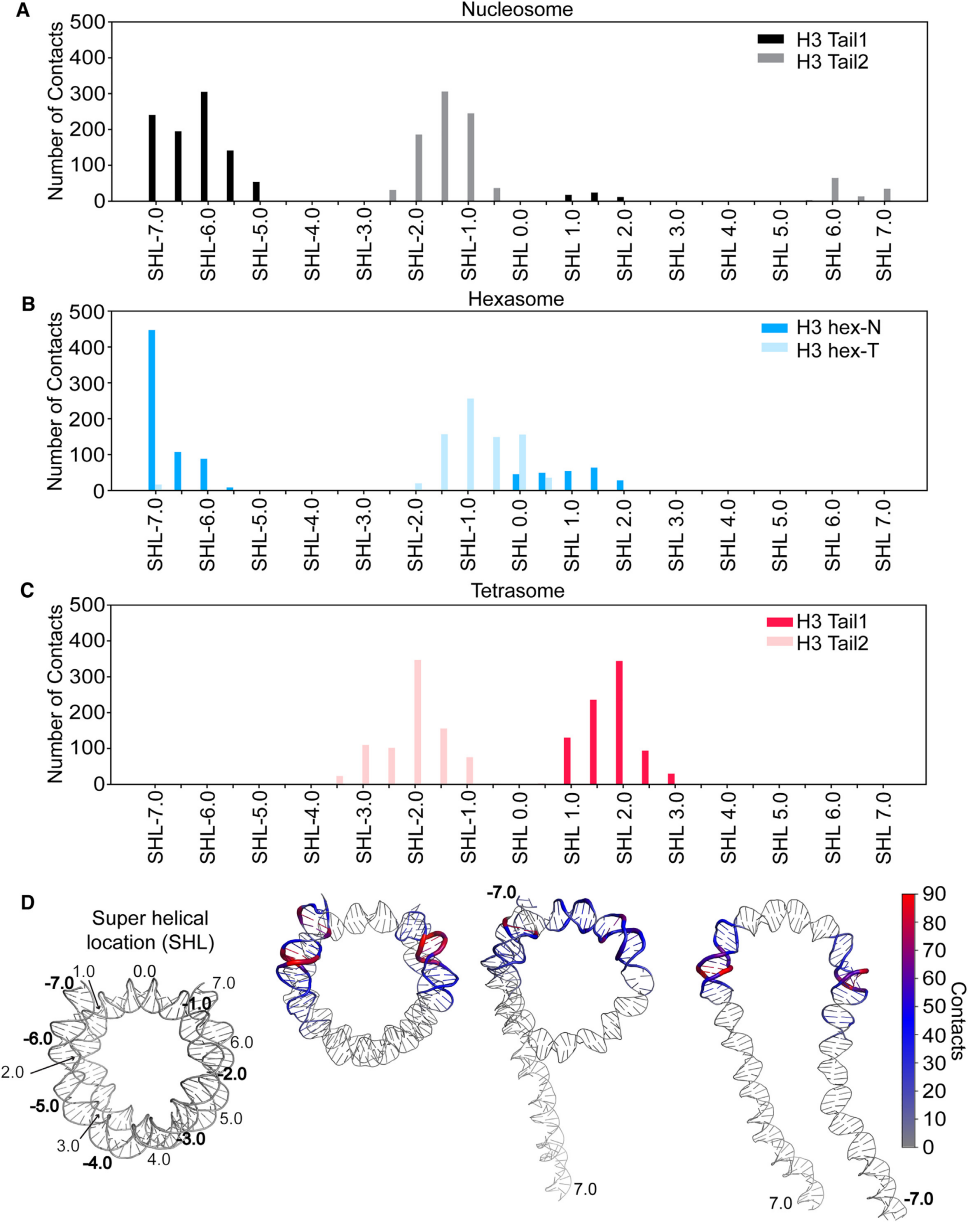
H3 tail contacts with DNA super helical locations (SHLs) in nucleosomal and subnucleosomal species. Plots are for (**A**) nucleosome, (**B**) hexasome, and (**C**) tetrasome. Contacts are summed over each SHL and plotted for each H3 tail. (**D**) The number of contacts formed between H3 tail residues and DNA base-pairs were mapped onto nucleosomal and subnucleosomal DNA. The thickness and color (see key) of the cartoon backbone represents the number of contacts for a given base pair, where thicker indicates more contacts. A representative end-state structure is used for each species. Histones are omitted from the image for ease of visualization.

To further quantify the conformational states of the H3 tails, contacts between the tails and DNA super helical locations (SHL) were calculated (Figure 5). For the nucleosome, the tails are seen to bind on either side of the dyad (SHLs –2.5 to 2.0) and outer DNA turns (SHL –7.0 to SHL –5.0 and SHL 6.0 to SHL 7.0). Notably, this positioning is in agreement with a cross-linking study that found contacts between the H3 tail (probe placed at H3T6C or H3A15C) and SHLs ±1.5 and ±2.0 in nucleosomes formed with 207 bp–601 DNA (43). (Interestingly, this folding back of the tail to interact with a range of locations on the core DNA was observed even though linker DNA was present.) In comparison, the tetrasomal H3 tails extend away from the dyad, binding at SHLs –3.5 to 3.0, without making any contacts with SHL 0.0. This could be due to the DNA unwrapping, facilitating additional SHL contacts more distant from the dyad, and is consistent with a more extended conformation (Supplementary Figure S12). For the hexasome, the hex-N tail is similar to a nucleosomal conformation, forming contacts with inner and outer DNA turn around SHL –7.0 to –5.5, and SHL 0 to 2.0. In contrast, the hex-T tail adopts unique contacts with SHL –2.0 to 0.5, occupying the region on and near the dyad and even extending across the dyad to make some cross-gyre interactions (SHL –7.0) with the wrapped DNA. This is again consistent with a more extended conformation (Supplementary Figure S12). Notably, the loss of any contacts with SHL –7.0 to –5.5 is consistent with the previous observation that in hexasomes the unwrapped DNA is as accessible as naked DNA to transcription factor binding, indicating no competition with histone tails for binding to this DNA (27). Of all species, the nucleosome tails have the highest number of contacts per base-pair, which are comparable to the number of contacts in the hex-N tail (Table 1). In contrast, the hex-T tail forms the fewest number of contacts with the DNA (while still overall in a DNA-bound state).

**Table 1.** Summary of binding energetics, solvent exposed surface area, and contacts of individual H3 tails from molecular dynamics simulations

H3 tails	System	Δ*E*_DNA-tail_ (kcal/mol)	Solvent accessible surface area (Å^2^)	Total number of contacts
Tail1	Nucleosome	–131.0±1.7	2103.2 ± 48.0	922
	Hexasome (hex-N)	–123.3±2.0	1989.3 ± 52.7	891
	Tetrasome	–119.0±2.4	2313.2 ± 64.3	815
Tail2	Nucleosome	–132.0±1.6	2022.7 ± 48.7	989
	Hexasome (hex-T)	–110.5±1.5	2210.9 ± 48.6	789
	Tetrasome	–116.0±1.3	2327.9 ± 37.8	837

To analyze the interaction energetics of these H3 tail/DNA conformations, an MM/GBSA analysis was performed for residues 1–37 of the H3 tail. Nucleosomal H3 tails bound to DNA with similar energies of –131.0 ± 1.7 kcal/mol and –132.1 ± 1.6 kcal/mol, respectively (Table 1). In comparison the tetrasomal H3 tails bound to DNA weaker with energies of –119.0 ± 2.4 kcal/mol and –116 ± 1.3 kcal/mol, which are not statistically significantly different (*P* > 0.2) from each other. In the hexasome, the hex-T tail bound with a statistically significantly (*P* < 0.0001) lower energy than the hex-N tail at –110.5 ± 1.5 kcal/mol and –123.3 ± 2.0 kcal/mol, respectively. These weaker H3 tail binding affinities upon H2A/H2B dimer loss are accompanied by greater solvent exposure of these residues. This was determined from the calculated solvent accessible surface area (SASA), which is ∼250 Å^2^ greater in the tetrasomal and hex-T tails as compared to the nucleosomal and hex-N tails. We note that care must be taken when interpreting MM/GBSA results, as it involves several approximations. These include a mean-field solvent and the lack of configurational entropy calculations, which is likely significantly different between the tails as demonstrated by RMSF calculations. Therefore, these energies should be interpreted only qualitatively.

Altogether, the MD simulations are consistent with the NMR data, where upon loss of the H2A/H2B dimer and unwrapping of the DNA, the H3 tail remains in a DNA bound state. However, the tails experience increased conformational dynamics with respect to the core. In the hexasome, this leads to asymmetric conformational ensembles and dynamics of the two H3 tails.

### The H3 tail has differential accessibility between nucleosomal species

To test whether the increase in dynamics and change in conformational ensemble of the H3 tail upon H2A/H2B dimer loss leads to increased accessibility, we performed trypsin proteolysis in the context of the nucleosome, hexasome and tetrasome. Trypsin proteolysis is a natural mode of tail clipping (73) and serves as a useful method to probe the general accessibility of the histone tails in a relatively non-sequence-specific manner because trypsin preferentially cleaves on the C-terminal side of lysine and arginine residues, which are spread out along the length of the tail (74,75).

**Figure 6.**
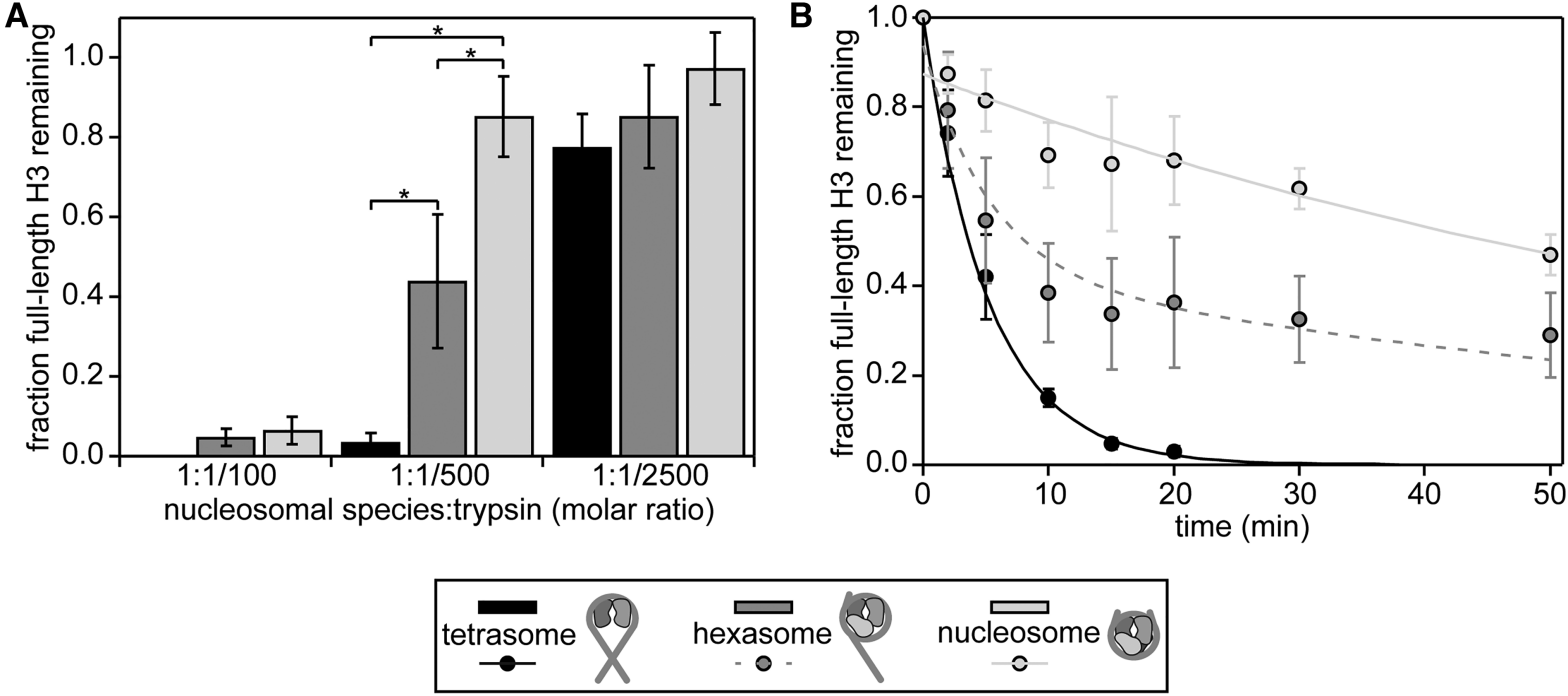
Trypsin digestion assays support differential accessibility of the H3 tail within different nucleosomal species. Gel-based trypsin digestion assays were used to probe tail accessibility. (**A**) The bar graph displays the results from proteolysis at a constant concentration of nucleosomal species (3 μM) and varying concentrations of trypsin. The progress of the trypsin proteolysis was assessed via SDS-PAGE. The fraction of full-length H3 remaining at *t* = 20 min as compared to *t* = 0 is shown, as determined by the intensities (volumes) of gel bands for full-length H3. The average and standard deviation are depicted from three experimental replicates. Data are marked (*) if differences between nucleosomal species at a given trypsin concentration were determined to be statistically significant as determined by a two-way ANOVA followed by a tukey post-hoc analysis (*P* <0.05). (**B**) The time course of a trypsin digestion was followed at the 1:1/500 molar ratio of nucleosomal species:trypsin with 3 μM of the given nucleosomal species. The progress of trypsin proteolysis was assessed via SDS-PAGE. The fraction of full-length H3 remaining is plotted as a function of time. The intensities (volumes) of gel bands for full-length H3 were normalized to H3 at *t* = 0, and the average and standard deviation are depicted from three gel replicates. Weighted single exponential fits (constrained to decay to zero and to have *y*-intercept ≤ 1) are shown for nucleosome and tetrasome (solid lines). The sum of the two exponential decays, with each weighted by one-half, represents the predicted time course for hexasome (dashed line).

Each nucleosomal species was incubated for 20 minutes with three different amounts of trypsin (1:1/100, 1:1/500 and 1:1/2500 molar ratio of nucleosomal species:trypsin). The amount of proteolysis was ascertained via SDS-PAGE (Figure 6A and Supplementary Figure S14A) by monitoring the amount of full-length H3 remaining. Note that since trypsin proteolysis can occur at multiple sites on the tails and we quantify the fraction of full length H3 remaining, this is a measure of the overall accessibility. In addition, the signal represents a sum of both H3s within each sample, since the two tails cannot be distinguished. Plotting the amount of full-length H3 remaining for each ratio of trypsin tested (Figure 6A) shows that each species undergoes different levels of proteolysis. The general trend indicates substantially greater proteolysis of the tetrasomal H3 tails as compared to the nucleosomal H3 tails. Notably, the level of proteolysis of the hexasomal H3 tails lies in between the nucleosome and tetrasome.

To further quantify the H3 tail accessibility to trypsin digest, we acquired kinetic time courses at a ratio of nucleosomal species:trypsin of 1:1/500 (Figure 6B and Supplementary Figure S14B). Experiments were conducted in triplicate, and the data were analyzed using a weighted fit of the fraction of remaining full-length H3 to a single exponential, similar to in (37,47) (see Materials and Methods for additional details). Importantly, native PAGE confirms that the nucleosomal species remained largely intact during the experiments (Supplementary Figure S14B). However, data was fit allowing for an initial offset on the y-axis to account for any small population of nucleosomes that may have fallen apart during the rapid mixing at the beginning of the digestion as is done in restriction enzyme digestions experiments (47). The single-exponential fits imply cleavage rates of *k*_obs_ = 0.012 ± 0.002 min^−1^ for nucleosome and *k*_obs_ = 0.19 ± 0.02 min^−1^ for tetrasome (Figure 6B, solid light-grey and black lines, respectively). Under the conditions that the digestion rate is first order in enzyme (trypsin) concentration, which is supported by Figure 6A and Supplementary Figure S14A (lower left), the rate of digestion is proportional to the H3 tail site exposure equilibrium constant. This is the relative concentration of H3 tail accessible states as compared to inaccessible states. This overall approach is analogous to the studies that use restriction enzyme to measure DNA accessibility with partially unwrapped nucleosomes (48). From the ratio of *k*_obs_, the relative site exposure probability is calculated as 15.8 ± 0.3, implying that the accessibility of the H3 tails is an order of magnitude greater upon loss of the H2A/H2B dimers. If the hexasome consists of one nucleosomal H3 tail and one tetrasomal H3 tail, as expected from the NMR data, the hexasome time course should be a sum of the two exponential decays, with each weighted by one-half (Figure 6B, dashed medium-grey line). Indeed, the experimental data for the hexasome are in very close agreement with this predicted time course (*P* = 0.999 in a two-sample t-test), strongly supporting that one H3 tail is in the nucleosomal state and the other H3 tail is in the tetrasomal state.

These proteolysis studies suggest that loss of the H2A/H2B dimer leads to increased accessibility to H3 tail binding proteins due to a change in the conformational ensemble and/or dynamics of the tails. In addition, they support results from NMR and MD analysis that the hexasome contains one tail in a nucleosomal state and one tail in a tetrasomal state.

## DISCUSSION

In this study, we find that the conformational ensembles and accessibility of the histone H3 tails are modulated by nucleosome composition. NMR and MD provide complementary views of the H3 tails. Both analyses support that the H3 tails adopt distinct DNA-bound conformational ensembles between nucleosomal and subnucleosomal species. In particular, MD simulations support that loss of the H2A/H2B dimer results in the adjacent H3 tail sampling unique SHLs along the DNA and adopting more extended conformations. The NMR experiments support an increase in internal dynamics of the H3 tail on the ps-ns timescale, suggesting faster transitions between DNA bound states. While MD simulations also support that loss of the H2A/H2B dimer leads to greater dynamics of the adjacent H3 tail, these greater fluctuations are largely coupled to an increase in DNA dynamics rather than an increase in internal motions on the DNA on the current timescale. We speculate that this discrepancy may be due to the water model used in the MD simulations. Specifically, the standard TIP3P model used here may over-stabilize the tail/DNA interactions of each ensemble state and therefore suppress the localized dynamics on the ps-ns timescale. Indeed, this effect has recently been observed in the context of histone tail dynamics (76,77). Notably however, the tails are bound more weakly to the DNA and have greater solvent exposure upon loss of the dimer in these simulations in line with the NMR data.

**Figure 7.**
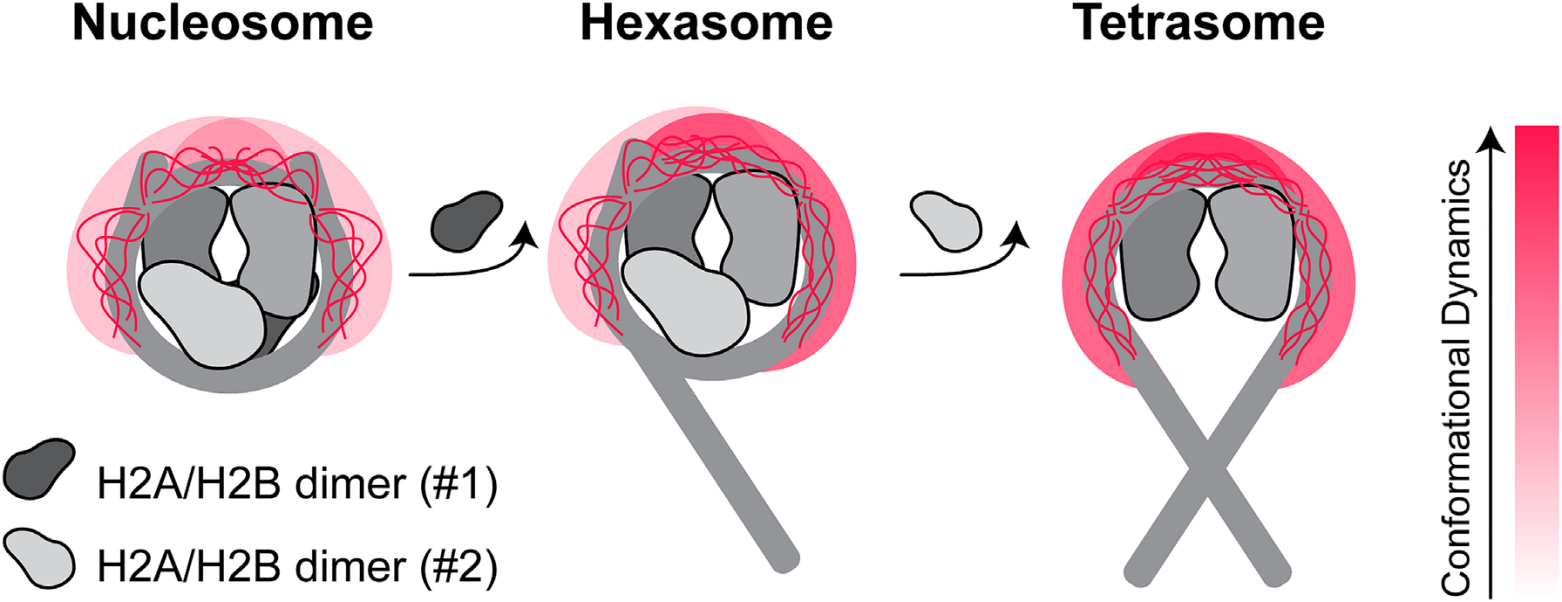
Model for the effect of nucleosome assembly state on H3 tail conformational ensemble. This cartoon model illustrates that loss of H2A/H2B dimer influences the conformational ensembles and dynamics of the adjacent H3 tail. DNA, H3/H4 and H2A/H2B dimers are shown in shades of grey. Of the histone tails, only the H3 tails are explicitly represented. The cartoon depicts a cloud for the tail conformational ensemble and explicitly depicts a subset of states within the DNA-bound ensemble. The shift in the color of the cloud from light to dark red indicates an increase in conformational dynamics within the ensemble. Shown from left to right are the nucleosome, hexasome (loss of one H2A/H2B dimer), and tetrasome (loss of both H2A/H2B dimers).

From these results, we propose a model wherein H2A/H2B dimer loss and concomitant DNA unwrapping lead to an H3 tail ensemble of DNA-bound states that samples more extended conformations with faster conformational dynamics (Figure 7). This is likely due to a change in the density of DNA around the tail upon DNA unwrapping. In the hexasome, this leads to asymmetric H3 tails in which one tail adopts a nucleosomal-like state and one tail a tetrasomal-like state. Importantly, these changes in conformational dynamics lead to increased accessibility of the tail, indicating that nucleosome composition can regulate histone tail signaling.

These results are in agreement with recent fluorescence studies that observed an increase in H3 tail dynamics upon salt-induced loss of dimer to form hexasome (25). Another recent study showed that replacement of DNA at one entry-exit site by the pAID of FACT, which also breaks the pseudo-symmetry of the nucleosome similar to in the hexasome, leads to asymmetry of the conformation and dynamics of the two H3 tails within the nucleosome (72). In addition, the results presented here are reminiscent of recent studies which found that binding of HMGN1 and HMGN2 to nucleosomes shift the location of H3 tail-DNA contacts, which was proposed to be involved in modulating chromatin condensation (43). Thus, modulating the conformational ensemble of the H3 tail within the nucleosome (or its sub-species) may be a general mechanism for regulating chromatin structure and accessibility.

Previous studies addressing histone tail accessibility in the context of the nucleosome have shown that the tails are significantly occluded within the nucleosome as compared to histone peptides or refolded histones (30,35–37). In the case of the H3 tail, accessibility to chemical modification is reduced by a factor of ∼250 at 50 mM NaCl and ∼10 at 150 mM NaCl (30). It has been proposed that accessibility to the H3 tail could be modulated by a number of factors including histone PTMs and DNA dynamics. Here, we find that, in the absence of salt, accessibility of the nucleosomal H3 tail to trypsin is increased by a factor of ∼16 upon H2A/H2B dimer loss and increased tail dynamics. Notably, this is consistent with recent data showing that *in vivo* cleavage of the H3 tail by trypsins is enhanced by acetylation, which has also been shown to increase the dynamics of the H3 tail (36,73). These results also align with investigations into the linked PHD fingers of CHD4, which bind the H3 tails in an DNA-depleted 80 bp-tetrasome with greater affinity than the 147 bp-nucleosome (30). In addition, replacement of DNA at one entry-exit site by FACT pAID modulates accessibility of the H3 tail to acetylation by Gcn5 (72). Altogether, this supports that altering nucleosome composition, whether by H2A/H2B dimer loss or other modes of DNA displacement, will modulate accessibility of chromatin-associated proteins to the H3 tail.

The modulation of H3 tail conformational dynamics and accessibility seen here are intriguing in light of previously observed changes in DNA dynamics and accessibility. In particular, loss of the H2A/H2B dimer leads to DNA unwrapping and increased transcription factor association to an exposed consensus site to the level of free DNA (27). In the hexasome, unwrapping of one side stabilizes the still-wrapped side, decreasing the unwrapping dynamics and association of transcription factors to a consensus site as compared to a nucleosome (27). It has been hypothesized that this reduced DNA unwrapping is due to rearrangement of the histones upon loss of the dimer. Here, simulations suggest that in the hexasome the H3 tail of the still-wrapped side adopts a more compact conformation on the wrapped DNA. In addition, the H3 tail from the unwrapped side crosses the dyad to the still-wrapped side to make additional contacts with the wrapped DNA. Thus, the H3 tail may be aiding in the observed stabilization.

In addition, since loss of H2A/H2B dimer leads to both DNA unwrapping and site exposure, as well as increased H3 tail accessibility, these concomitant changes could function cooperatively. For example, a protein domain that binds transiently to partially unwrapped nucleosomal DNA is anticipated to increase the H3 tail accessibility to an H3 tail binding domain. This could result in cooperative binding similar to how adjacent transcription factor binding sites can result in cooperative binding (78). Furthermore, if the DNA and H3 tail binding domains are within the same protein or complex, the concomitant increase in accessibility of DNA and histone H3 tail could multiplicatively increase the binding probability. This could preferentially target complexes to the side of the hexasome that is missing the H2A/H2B dimer. Future studies will be needed to directly investigate these potential cooperativity mechanisms.

While recent data indicates the presence of sub-nucleosomes *in vivo*, their role is not yet fully understood. However, *in vitro* studies indicate that they modulate the activity of chromatin regulators (such as ATP-dependent remodelers) and RNA polymerase. In particular, the observed asymmetry of the hexasome has been hypothesized to play an important regulatory role. Here we observe that the histone H3 tail conformational dynamics and accessibility are regulated by the sub-nucleosome state and are asymmetric in the hexasome. We expect this will modulate the activity of chromatin modifiers and ATP-dependent remodelers, helping to shape the chromatin landscape, and may also contribute to regulation of transcription.

## DATA AVAILABILITY

Chemical shift data in different conditions and relaxation data are available in Supplementary Table S1. All additional raw data will be provided upon request. BMRB deposition numbers are 50805, 50806 and 50807 for ^13^C/^15^N-H3 tetrasome, nucleosome, and hexasome, respectively.

## Supplementary Material

gkab246_Supplemental_Files

## References

[1] Zlatanova J. , Bishop T.C., Victor J.-M., Jackson V., Holde K. The nucleosome family: dynamic and growing. Struct./Fold. Des. 2009; 17:160–171. 10.1016/j.str.2008.12.016 19217387

[2] Akey C.W. , Luger K. Histone chaperones and nucleosome assembly. Curr. Opin. Struct. Biol. 2003; 13:6–14. 12581654 10.1016/s0959-440x(03)00002-2

[3] Park Y.-J. , Luger K. Histone chaperones in nucleosome eviction and histone exchange. Curr. Opin. Struct. Biol. 2008; 18:282–289. 18534842 10.1016/j.sbi.2008.04.003 PMC2525571

[4] Gansen A. , Valeri A., Hauger F., Felekyan S., Kalinin S., Tóth K., Langowski J., Seidel C.A.M. Nucleosome disassembly intermediates characterized by single-molecule FRET. PNAS. 2009; 106:15308–15313. 19706432 10.1073/pnas.0903005106 PMC2741247

[5] Azegami N. , Saikusa K., Todokoro Y., Nagadoi A., Kurumizaka H., Nishimura Y., Akashi S. Conclusive evidence of the reconstituted hexasome proven by native mass spectrometry. Biochemistry. 2013; 52:5155–5157. 23879667 10.1021/bi4005655

[6] Gansen A. , Felekyan S., Kühnemuth R., Lehmann K., Tóth K., Seidel C.A.M., Langowski J. High precision FRET studies reveal reversible transitions in nucleosomes between microseconds and minutes. Nat. Commun. 2018; 9:4628. 30401903 10.1038/s41467-018-06758-1 PMC6219519

[7] Mattiroli F. , Gu Y., Yadav T., Balsbaugh J.L., Harris M.R., Findlay E.S., Liu Y., Radebaugh C.A., Stargell L.A., Ahn N.G. et al. DNA-mediated association of two histone-bound complexes of yeast Chromatin Assembly Factor-1 (CAF-1) drives tetrasome assembly in the wake of DNA replication. eLife. 2017; 6:e22799. 28315523 10.7554/eLife.22799 PMC5404915

[8] Wang T. , Liu Y., Edwards G., Krzizike D., Scherman H., Luger K. The histone chaperone FACT modulates nucleosome structure by tethering its components. Life Sci. Alliance. 2018; 1:e201800107. 30456370 10.26508/lsa.201800107 PMC6238592

[9] Pardal A.J. , Fernandes-Duarte F., Bowman A.J. The histone chaperoning pathway: from ribosome to nucleosome. Essays Biochem. 2019; 63:29–43. 31015382 10.1042/EBC20180055 PMC6484783

[10] Kireeva M.L. , Walter W., Tchernajenko V., Bondarenko V., Kashlev M., Studitsky V.M. Nucleosome remodeling induced by RNA polymerase II: loss of the H2A/H2B dimer during transcription. Mol. Cell. 2002; 9:541–552. 11931762 10.1016/s1097-2765(02)00472-0

[11] Levchenko V. , Jackson B., Jackson V. Histone release during transcription: displacement of the two H2A-H2B dimers in the nucleosome is dependent on different levels of transcription-induced positive stress. Biochemistry. 2005; 44:5357–5372. 15807529 10.1021/bi047786o

[12] Dechassa M.L. , Sabri A., Pondugula S., Kassabov S.R., Chatterjee N., Kladde M.P., Bartholomew B. SWI/SNF has intrinsic nucleosome disassembly activity that is dependent on adjacent nucleosomes. Mol. Cell. 2010; 38:590–602. 20513433 10.1016/j.molcel.2010.02.040 PMC3161732

[13] Prasad R. , D’Arcy S., Hada A., Luger K., Bartholomew B. Coordinated action of Nap1 and RSC in disassembly of tandem nucleosomes. Mol. Cell. Biol. 2016; 36:2262–2271. 27273866 10.1128/MCB.00195-16 PMC4985928

[14] Kulaeva O.I. , Gaykalova D.A., Pestov N.A., Golovastov V.V., Vassylyev D.G., Artsimovitch I., Studitsky V.M. Mechanism of chromatin remodeling and recovery during passage of RNA polymerase II. Nat. Struct. Mol. Biol. 2009; 16:1272–1278. 19935686 10.1038/nsmb.1689 PMC2919570

[15] Levendosky R.F. , Sabantsev A., Deindl S., Bowman G.D. The Chd1 chromatin remodeler shifts hexasomes unidirectionally. eLife. 2016; 5:e21356. 28032848 10.7554/eLife.21356 PMC5226652

[16] Qiu Y. , Levendosky R.F., Chakravarthy S., Patel A., Bowman G.D., Myong S. The Chd1 chromatin remodeler shifts nucleosomal DNA bidirectionally as a monomer. Mol. Cell. 2017; 68:76–88. 28943314 10.1016/j.molcel.2017.08.018 PMC5745159

[17] Rhee H.S. , Bataille A.R., Zhang L., Pugh B.F. Subnucleosomal structures and nucleosome asymmetry across a genome. Cell. 2014; 159:1377–1388. 25480300 10.1016/j.cell.2014.10.054 PMC4258235

[18] Ramachandran S. , Ahmad K., Henikoff S. Transcription and remodeling produce asymmetrically unwrapped nucleosomal intermediates. Mol. Cell. 2017; 68:1038–1053. 29225036 10.1016/j.molcel.2017.11.015 PMC6421108

[19] Arimura Y. , Tachiwana H., Oda T., Sato M., Kurumizaka H. Structural analysis of the hexasome, lacking one histone H2A/H2B dimer from the conventional nucleosome. Biochemistry. 2012; 51:3302–3309. 22448809 10.1021/bi300129b

[20] Chen Y. , Tokuda J.M., Topping T., Sutton J.L., Meisburger S.P., Pabit S.A., Gloss L.M., Pollack L. Revealing transient structures of nucleosomes as DNA unwinds. Nucleic Acids Res. 2014; 42:8767–8776. 24990379 10.1093/nar/gku562 PMC4117781

[21] Nazarov I. , Chekliarova I., Rychkov G., Ilatovskiy A.V., Crane-Robinson C., Tomilin A. AFM studies in diverse ionic environments of nucleosomes reconstituted on the 601 positioning sequence. Biochimie. 2016; 121:5–12. 26586109 10.1016/j.biochi.2015.11.010

[22] Rychkov G.N. , Ilatovskiy A.V., Nazarov I.B., Shvetsov A.V., Lebedev D.V., Konev A.Y., Isaev-Ivanov V.V., Onufriev A.V. Partially assembled nucleosome structures at atomic detail. Biophys. J. 2017; 112:460–472. 28038734 10.1016/j.bpj.2016.10.041 PMC5300784

[23] Chen Y. , Tokuda J.M., Topping T., Meisburger S.P., Pabit S.A., Gloss L.M., Pollack L. Asymmetric unwrapping of nucleosomal DNA propagates asymmetric opening and dissociation of the histone core. PNAS. 2017; 114:334–339. 28028239 10.1073/pnas.1611118114 PMC5240728

[24] Matsumoto A. , Sugiyama M., Li Z., Martel A., Porcar L., Inoue R., Kato D., Osakabe A., Kurumizaka H., Kono H. Structural studies of overlapping dinucleosomes in solution. Biophys. J. 2019; 118:2209–2219. 31952809 10.1016/j.bpj.2019.12.010 PMC7202943

[25] Lehmann K. , Felekyan S., Kühnemuth R., Dimura M., Tóth K., Seidel C.A.M., Langowski J. Dynamics of the nucleosomal histone H3 N-terminal tail revealed by high precision single-molecule FRET. Nucleic Acids Res. 2020; 319:1097–1021. 10.1093/nar/gkz1186 PMC7026643 31956896

[26] Kato D. , Osakabe A., Arimura Y., Mizukami Y., Horikoshi N., Saikusa K., Akashi S., Nishimura Y., Park S.-Y., Nogami J. et al. Crystal structure of the overlapping dinucleosome composed of hexasome and octasome. Science. 2017; 356:205–208. 28408607 10.1126/science.aak9867

[27] Brehove M. , Shatoff E., Donovan B.T., Jipa C.M., Bundschuh R., Poirier M.G. DNA sequence influences hexasome orientation to regulate DNA accessibility. Nucleic Acids Res. 2019; 47:5617–5633. 31216039 10.1093/nar/gkz283 PMC6582347

[28] Mutskov V. , Gerber D., Angelov D., Ausio J., Workman J., Dimitrov S. Persistent interactions of core histone tails with nucleosomal DNA following acetylation and transcription factor binding. Mol. Cell. Biol. 1998; 18:6293–6304. 9774646 10.1128/mcb.18.11.6293 PMC109216

[29] Pilotto S. , Speranzini V., Tortorici M., Durand D., Fish A., Valente S., Forneris F., Mai A., Sixma T.K., Vachette P. et al. Interplay among nucleosomal DNA, histone tails, and corepressor CoREST underlies LSD1-mediated H3 demethylation. PNAS. 2015; 112:2752–2757. 25730864 10.1073/pnas.1419468112 PMC4352788

[30] Gatchalian J. , Wang X., Ikebe J., Cox K.L., Tencer A.H., Zhang Y., Burge N.L., Di L., Gibson M.D., Musselman C.A. et al. Accessibility of the histone H3 tail in the nucleosome for binding of paired readers. Nat. Commun. 2017; 8:1489. 29138400 10.1038/s41467-017-01598-x PMC5686127

[31] Kan P.Y. , Lu X., Hansen J.C., Hayes J.J. The H3 tail domain participates in multiple interactions during folding and self-association of nucleosome arrays. Mol. Cell. Biol. 2007; 27:2084–2091. 17242202 10.1128/MCB.02181-06 PMC1820515

[32] Shaytan A.K. , Armeev G.A., Goncearenco A., Zhurkin V.B., Landsman D., Panchenko A.R. Coupling between histone conformations and DNA geometry in nucleosomes on a microsecond timescale: atomistic insights into nucleosome functions. J. Mol. Biol. 2016; 428:221–237. 26699921 10.1016/j.jmb.2015.12.004 PMC4738025

[33] Li Z. , Kono H. Distinct roles of histone H3 and H2A tails in nucleosome stability. Sci. Rep. 2016; 6:srep31437. 10.1038/srep31437 PMC4985630 27527579

[34] Furukawa A. , Wakamori M., Arimura Y., Ohtomo H., Tsunaka Y., Kurumizaka H., Umehara T., Nishimura Y. Acetylated histone H4 tail enhances histone H3 tail acetylation by altering their mutual dynamics in the nucleosome. Proc. Natl. Acad. Sci. U.S.A. 2020; 117:19661–19663. 32747537 10.1073/pnas.2010506117 PMC7443954

[35] Morrison E.A. , Bowerman S., Sylvers K.L., Wereszczynski J., Musselman C.A. The conformation of the histone H3 tail inhibits association of the BPTF PHD finger with the nucleosome. eLife. 2018; 7:e78587. 10.7554/eLife.31481 PMC5953545 29648537

[36] Stützer A. , Liokatis S., Kiesel A., Schwarzer D., Sprangers R., Söding J., Selenko P., Fischle W. Modulations of DNA contacts by linker histones and post-translational modifications determine the mobility and modifiability of nucleosomal H3 Tails. Mol. Cell. 2016; 61:247–259. 26778125 10.1016/j.molcel.2015.12.015

[37] Wang X. , Hayes J.J. Site-specific binding affinities within the H2B tail domain indicate specific effects of lysine acetylation. J. Biol. Chem. 2007; 282:32867–32876. 17711854 10.1074/jbc.M706035200

[38] Dyson H.J. Roles of intrinsic disorder in protein–nucleic acid interactions. Mol. Biosyst. 2012; 8:97–104. 21874205 10.1039/c1mb05258f PMC3600845

[39] Tompa P. , Fuxreiter M. Fuzzy complexes: polymorphism and structural disorder in protein–protein interactions. Trends Biochem. Sci. 2008; 33:2–8. 18054235 10.1016/j.tibs.2007.10.003

[40] Borgia A. , Borgia M.B., Bugge K., Kissling V.M., Heidarsson P.O., Fernandes C.B., Sottini A., Soranno A., Buholzer K.J., Nettels D. et al. Extreme Disorder in an Ultrahigh-affinity Protein Complex. Nature. 2018; 555:61–66. 29466338 10.1038/nature25762 PMC6264893

[41] Fuxreiter M. Fuzziness in protein interactions—a historical perspective. J. Mol. Biol. 2018; 430:2278–2287. 29477337 10.1016/j.jmb.2018.02.015

[42] Ghoneim M. , Fuchs H.A., Musselman C.A. Histone tail conformations: a fuzzy affair with DNA. Trends Biochem. Sci. 2021; doi:10.1016/j.tibs.2020.12.012. 10.1016/j.tibs.2020.12.012 PMC8195839 33551235

[43] Murphy K.J. , Cutter A.R., Fang H., Postnikov Y.V., Bustin M., Hayes J.J. HMGN1 and 2 remodel core and linker histone tail domains within chromatin. Nucleic Acids Res. 2017; 45:9917–9930. 28973435 10.1093/nar/gkx579 PMC5622319

[44] Dyer P.N. , Edayathumangalam R.S., White C.L., Bao Y., Chakravarthy S., Muthurajan U.M., Luger K. Reconstitution of nucleosome core particles from recombinant histones and DNA. Methods Enzymol. 2004; 375:23–44. 14870657 10.1016/s0076-6879(03)75002-2

[45] Delaglio F. , Grzesiek S., Vuister G.W., Zhu G., Pfeifer J., Bax A. NMRPipe: a multidimensional spectral processing system based on UNIX pipes. J. Biomol. NMR. 1995; 6:277–293. 8520220 10.1007/BF00197809

[46] Vranken W.F. , Boucher W., Stevens T.J., Fogh R.H., Pajon A., Llinas M., Ulrich E.L., Markley J.L., Ionides J., Laue E.D. The CCPN data model for NMR spectroscopy: development of a software pipeline. Proteins. 2005; 59:687–696. 15815974 10.1002/prot.20449

[47] Polach K.J. , Widom J. Restriction enzymes as probes of nucleosome stability and dynamics. Methods Enzymol. 1999; 304:278–298. 10372366 10.1016/s0076-6879(99)04017-3

[48] Polach K.J. , Widom J. Mechanism of protein access to specific DNA sequences in chromatin: a dynamic equilibrium model for gene regulation. J. Mol. Biol. 1995; 254:130–149. 7490738 10.1006/jmbi.1995.0606

[49] Poirier M.G. , Bussiek M., Langowski J., Widom J. Spontaneous access to DNA target sites in folded chromatin fibers. J. Mol. Biol. 2008; 379:772–786. 18485363 10.1016/j.jmb.2008.04.025 PMC2481406

[50] Makde R.D. , England J.R., Yennawar H.P., Tan S. Structure of RCC1 chromatin factor bound to the nucleosome core particle. Nature. 2010; 467:562–566. 20739938 10.1038/nature09321 PMC3168546

[51] Davey C.A. , Sargent D.F., Luger K., Maeder A.W., Richmond T.J. Solvent mediated interactions in the structure of the nucleosome core particle at 1.9 a resolution. J. Mol. Biol. 2002; 319:1097–1113. 12079350 10.1016/S0022-2836(02)00386-8

[52] Shen M.-Y. , Sali A. Statistical potential for assessment and prediction of protein structures. Protein Sci. 2006; 15:2507–2524. 17075131 10.1110/ps.062416606 PMC2242414

[53] Salomon-Ferrer R. , Götz A.W., Poole D., Grand S.L., Walker R.C. Routine microsecond molecular dynamics simulations with AMBER on GPUs. 2. Explicit solvent particle mesh Ewald. J. Chem. Theory Comput. 2013; 9:3878–3888. 26592383 10.1021/ct400314y

[54] Grand S.L. , Götz A.W., Walker R.C. SPFP: Speed without compromise—a mixed precision model for GPU accelerated molecular dynamics simulations. Comput. Phys. Commun. 2013; 184:374–380.

[55] Maier J.A. , Martinez C., Kasavajhala K., Wickstrom L., Hauser K.E., Simmerling C. ff14SB: improving the accuracy of protein side chain and backbone parameters from ff99SB. J. Chem. Theory Comput. 2015; 11:3696–3713. 26574453 10.1021/acs.jctc.5b00255 PMC4821407

[56] Ivani I. , Dans P.D., Noy A., Pérez A., Faustino I., Hospital A., Walther J., Andrio P., Goñi R., Balaceanu A. et al. Parmbsc1: a refined force field for DNA simulations. Nat. Methods. 2016; 13:55–58. 26569599 10.1038/nmeth.3658 PMC4700514

[57] Nguyen H. , Roe D.R., Simmerling C. Improved generalized born solvent model parameters for protein simulations. J. Chem. Theory Comput. 2013; 9:2020–2034. 25788871 10.1021/ct3010485 PMC4361090

[58] Jorgensen W.L. , Chandrasekhar J., Madura J.D. Comparison of simple potential functions for simulating liquid water. J. Chem. Phys. 1983; 79:926–935.

[59] Joung I.S. , Cheatham T.E. Molecular dynamics simulations of the dynamic and energetic properties of alkali and halide ions using water-model-specific ion parameters. J. Phys. Chem. B. 2009; 113:13279–13290. 19757835 10.1021/jp902584c PMC2755304

[60] Hopkins C.W. , Grand S.L., Walker R.C., Roitberg A.E. Long-time-step molecular dynamics through hydrogen mass repartitioning. J. Chem. Theory Comput. 2015; 11:1864–1874. 26574392 10.1021/ct5010406

[61] Ryckaert J.P. , Ciccotti G., Berendsen H.J.C. Numerical integration of the cartesian equations of motion of a system with constraints: molecular dynamics of n-alkanes. J. Comput. Phys. 1977; 23:327–341.

[62] Loncharich R.J. , Brooks B.R., Pastor R.W. Langevin dynamics of peptides: the frictional dependence of isomerization rates of N-acetylalanyl-N’-methylamide. Biopolymers. 1992; 32:523–535. 1515543 10.1002/bip.360320508

[63] Humphrey W. , Dalke A., Schulten K. VMD: visual molecular dynamics. J. Mol. Graph. 1996; 14:33–38. 8744570 10.1016/0263-7855(96)00018-5

[64] DeLano W.L. The PyMOL molecular graphics system. 2002;

[65] Galindo-Murillo R. , Roe D.R., Cheatham T.E. Convergence and reproducibility in molecular dynamics simulations of the DNA duplex d(GCACGAACGAACGAACGC). Biochim. Biophys. Acta BBA - Gen. Subj. 2015; 1850:1041–1058. 10.1016/j.bbagen.2014.09.007 PMC4339415 25219455

[66] Roe D.R. , Cheatham T.E. PTRAJ and CPPTRAJ: software for processing and analysis of molecular dynamics trajectory data. J. Chem. Theory Comput. 2013; 9:3084–3095. 26583988 10.1021/ct400341p

[67] McClendon C.L. , Hua L., Barreiro A., Jacobson M.P. Comparing conformational ensembles using the Kullback-Leibler divergence expansion. J. Chem. Theory Comput. 2012; 8:2115–2126. 23316121 10.1021/ct300008d PMC3538811

[68] Michaud-Agrawal N. , Denning E.J., Woolf T.B., Beckstein O. MDAnalysis: a toolkit for the analysis of molecular dynamics simulations. J. Comput. Chem. 2011; 32:2319–2327. 21500218 10.1002/jcc.21787 PMC3144279

[69] Miller B.R. , McGee T.D., Swails J.M., Homeyer N., Gohlke H., Roitberg A.E. MMPBSA.py: an efficient program for end-state free energy calculations. J. Chem. Theory Comput. 2012; 8:3314–3321. 26605738 10.1021/ct300418h

[70] Ngo T.T.M. , Zhang Q., Zhou R., Yodh J.G., Ha T. Asymmetric unwrapping of nucleosomes under tension directed by DNA local flexibility. Cell. 2015; 160:1135–1144. 25768909 10.1016/j.cell.2015.02.001 PMC4409768

[71] Kay L.E. , Torchia D.A., Bax A. Backbone dynamics of proteins as studied by nitrogen-15 inverse detected heteronuclear NMR spectroscopy: application to staphylococcal nuclease. Biochemistry-us. 1989; 28:8972–8979. 10.1021/bi00449a003 2690953

[72] Tsunaka Y. , Ohtomo H., Morikawa K., Nishimura Y. Partial replacement of nucleosomal DNA with human FACT induces dynamic exposure and acetylation of histone H3 N-terminal tails. Iscience. 2020; 23:101641. 33103079 10.1016/j.isci.2020.101641 PMC7569332

[73] Ferrari K.J. , Amato S., Noberini R., Toscani C., Fernández-Pérez D., Rossi A., Conforti P., Zanotti M., Bonaldi T., Tamburri S. et al. Intestinal differentiation involves cleavage of histone H3 N-terminal tails by multiple proteases. Nucleic Acids Res. 2021; 49:791–804. 33398338 10.1093/nar/gkaa1228 PMC7826276

[74] Böhm L. , Crane-Robinson C. Proteases as structural probes for chromatin: the domain structure of histones. Biosci. Rep. 1984; 4:365–386. 6375755 10.1007/BF01122502

[75] Ausio J. , Dong F., Holde K.E. Use of selectively trypsinized nucleosome core particles to analyze the role of the histone “tails” in the stabilization of the nucleosome. J. Mol. Biol. 1989; 206:451–463. 2716057 10.1016/0022-2836(89)90493-2

[76] Rabdano S.O. , Shannon M.D., Izmailov S.A., Salguero N.G., Zandian M., Purusottam R.N., Poirier M.G., Skrynnikov N.R., Jaroniec C.P. Histone H4 tails in nucleosomes: a fuzzy interaction with DNA. Angew. Chem. Int. Ed. 2021; 60:6480–6487. 10.1002/anie.202012046 PMC7994933 33522067

[77] Peng Y. , Li S., Onufriev A., Landsman D., Panchenko A.R. Binding of regulatory proteins to nucleosomes is modulated by dynamic histone tails. 2020; bioRxiv doi:30 October 2020, preprint: not peer reviewed 10.1101/2020.10.30.360990. PMC8421395 34489435

[78] Adams C.C. , Workman J.L. Binding of disparate transcriptional activators to nucleosomal DNA is inherently cooperative. Mol. Cell. Biol. 1995; 15:1405–1421. 7862134 10.1128/mcb.15.3.1405 PMC230365

[79] Towns J. , Peterson G.D., Roskies R., Scott J.R., Wilkins-Diehr N., Cockerill T., Dahan M., Foster I., Gaither K., Grimshaw A. et al. XSEDE: accelerating scientific discovery. Comput. Sci. Eng. 2014; 16:62–74.

