# Supplementary material for: Nucleosome composition regulates the histone H3 tail conformational ensemble and accessibility
Source: Nucleic Acids Res. 2021 Apr 15;49(8):4750–67. doi: 10.1093/nar/gkab246 (PMC8096233; doi:10.1093/nar/gkab246)
Supplement: gkab246_Supplemental_Files [file gkab246_supplemental_files.zip › Supplementary_Figures.pdf]

Supplementary Figures

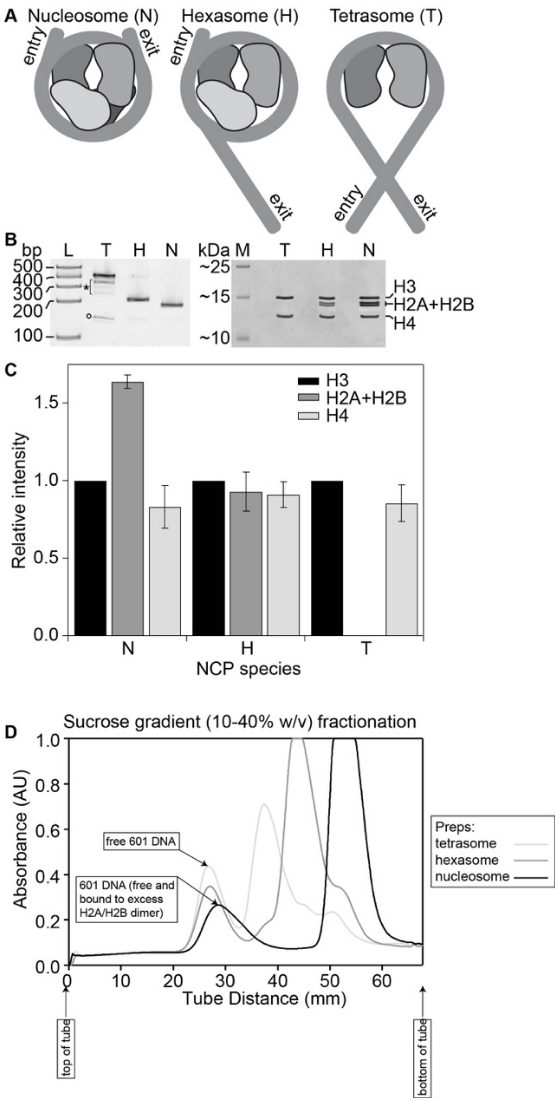

Supplementary Figure S1. Composition of nucleosomal species. **(A)** Cartoon depicting the composition of the three nucleosomal species investigated in this study, nucleosome, hexasome, and tetrasome. DNA and H3/H4 and H2A/H2B dimers are shown in shades of grey. Only the nucleosome core is represented. **(B)** Gels characterize the purified nucleosome (N), hexasome (H), and tetrasome (T). Native PAGE (5% acrylamide, left) and denaturing SDS-PAGE (18% acrylamide, right) confirm the identity of the species. The native gel was visualized with ethidium bromide and includes TrackIt 100bp DNA ladder (L) for size reference. The denaturing gel was stained with Coomassie and includes Spectra BR marker (M) for size reference. The asterisk (*) denotes putative alternative positioning of the tetrasome, and the circle (°) marks free 601 DNA. **(C)** The bar graph shows the relative intensities of the four histones within the three nucleosomal species as quantified from 18% denaturing acrylamide gels. The intensities (volumes) of gel bands were normalized to H3 to provide a relative intensity, and the average and standard deviation are depicted from four gel replicates. **(D)** Sucrose gradient purification of nucleosomes and subnucleosomes. The reconstitutions of nucleosomes and subnucleosomes (described in materials and methods) were purified via sucrose gradient (10-40% w/v sucrose). The major peak for each was used for experiments.

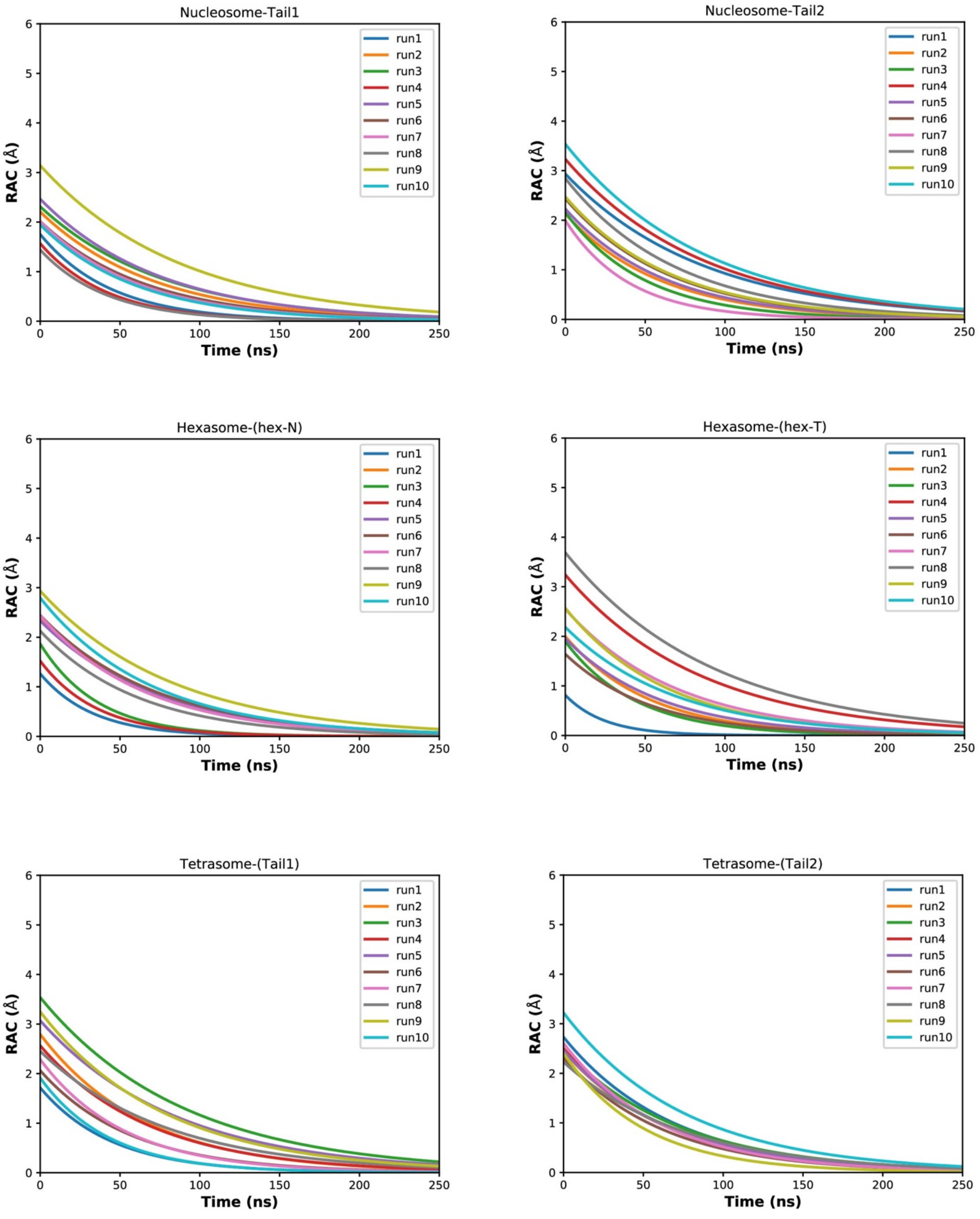

Supplementary Figure S2. RMS average correlation (RAC) of H3 tails computed at different time intervals using the ‘rmscorr’ command in the CPPTRAJ using the average structure from the trajectories as a reference for the calculations. Plots are for (top) nucleosome, (middle) hexasome, and (bottom) tetrasome.

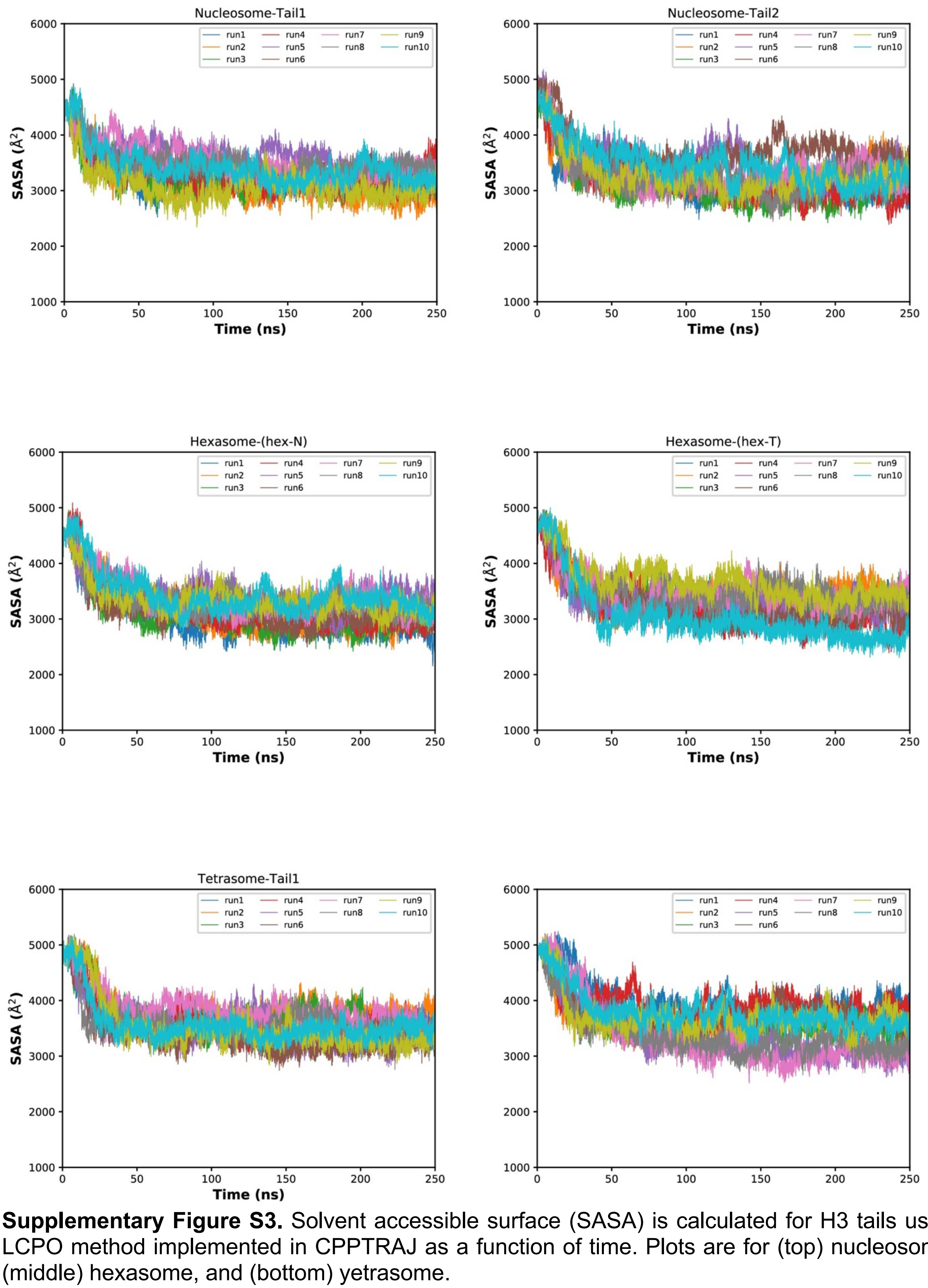

Supplementary Figure S3. Solvent accessible surface (SASA) is calculated for H3 tails using LCPO method implemented in CPPTRAJ as a function of time. Plots are for (top) nucleosome, (middle) hexasome, and (bottom) yetrasome.

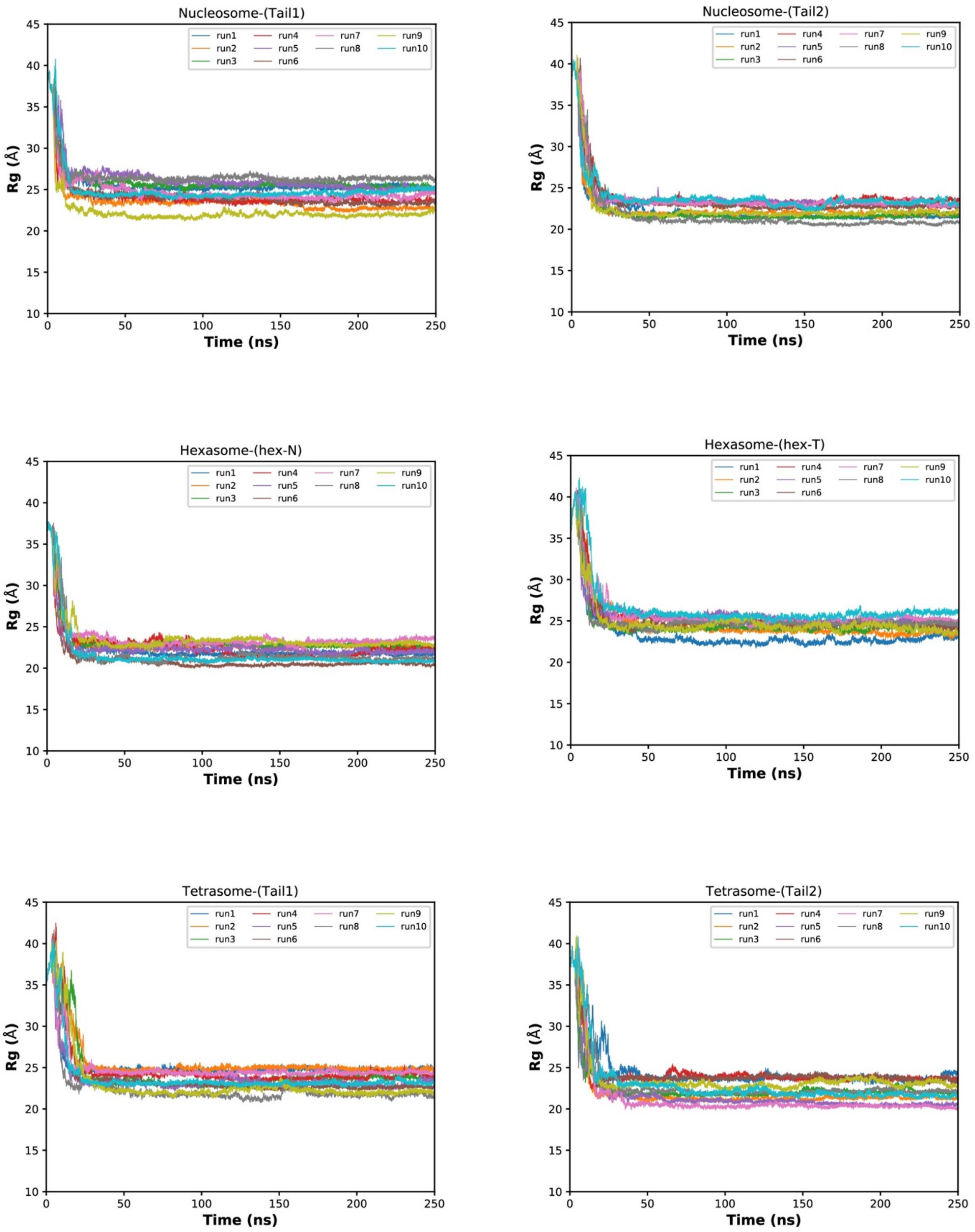

Supplementary Figure S4. Radius of gyration (Rg) of H3 tails as function of time from multiple simulations. Plots are for (top) nucleosome, (middle) hexasome, and (bottom) tetrasome.

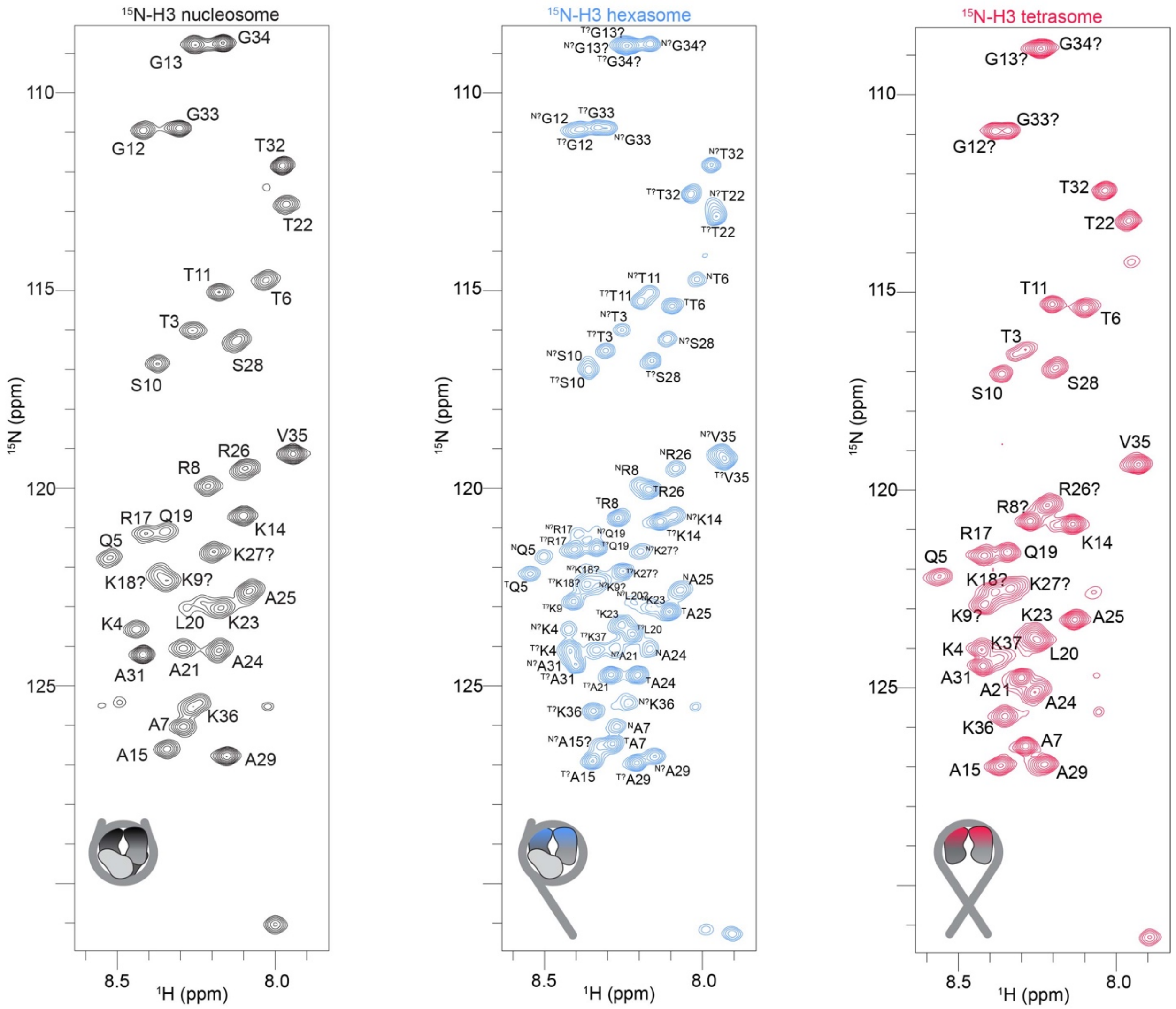

Supplementary Figure S5. Full-sized $^{1}H/^{15}N$-HSQC spectra of ^{15}N-H3-labeled nucleosome (black), hexasome (blue), and tetrasome (red) used in main text Figure 1. Peaks are labeled with residue assignments, and assignment uncertainty is indicated by '?' (see Table S1). For hexasome, hex-N and hex-T tail assignments are indicated by the super-script N and T designations (along with uncertainty status). These spectra were collected on 44μM ^{15}N-H3 nucleosomal species in 20mM MOPS pH 7, 1mM EDTA, 1mM DTT, 7% D2O at 37°C and on an 800MHz spectrometer.

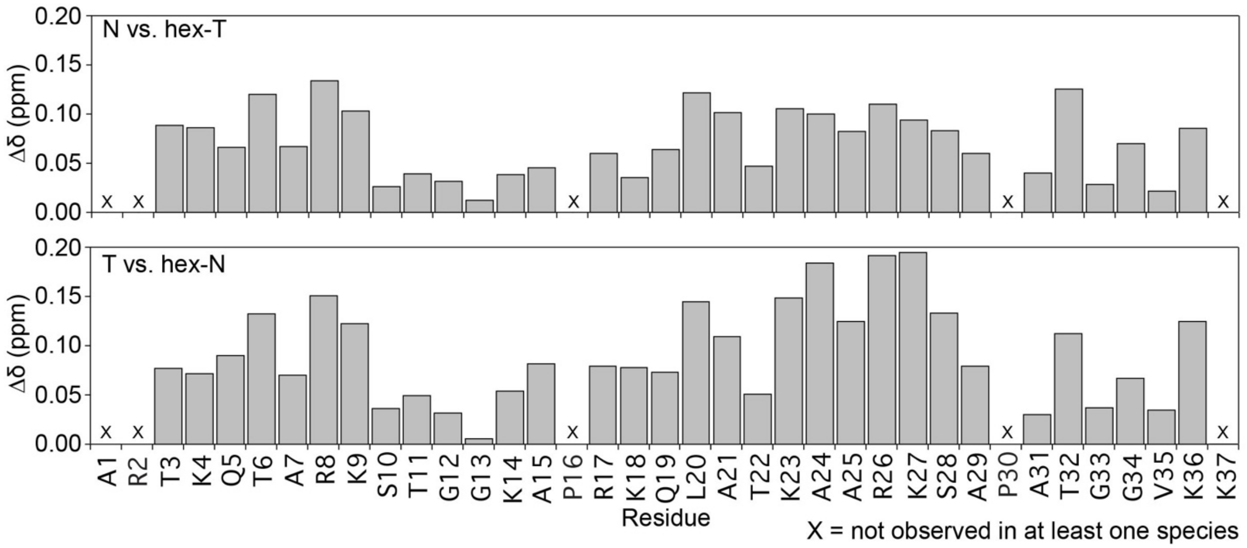

Supplementary Figure S6. Chemical shift differences (Δδ) between the nucleosome (N) and hex-T H3 tails (top) and the tetrasome (T) and hex-N H3 tails (bottom). These plots are shown as a function of H3 tail residue. Residues that are not observed in the spectrum of at least one species are marked with an 'X'.

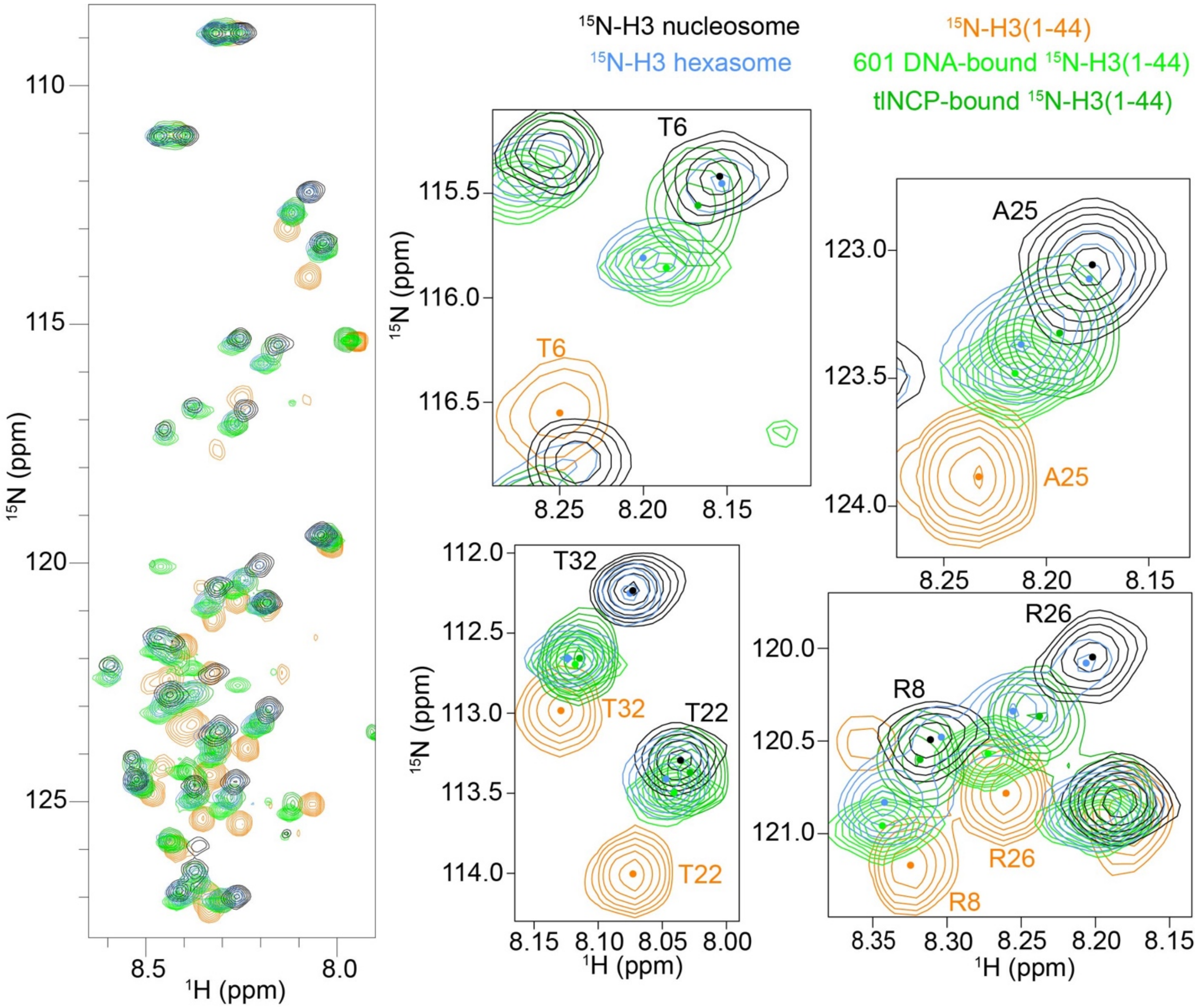

Supplementary Figure S7. Same as main text Figure 2, except that spectra are additionally overlaid that were collected on ^{15}N-H3(1-44) bound to either 601 DNA (lime green) or tailless NCP (green, from [32]).

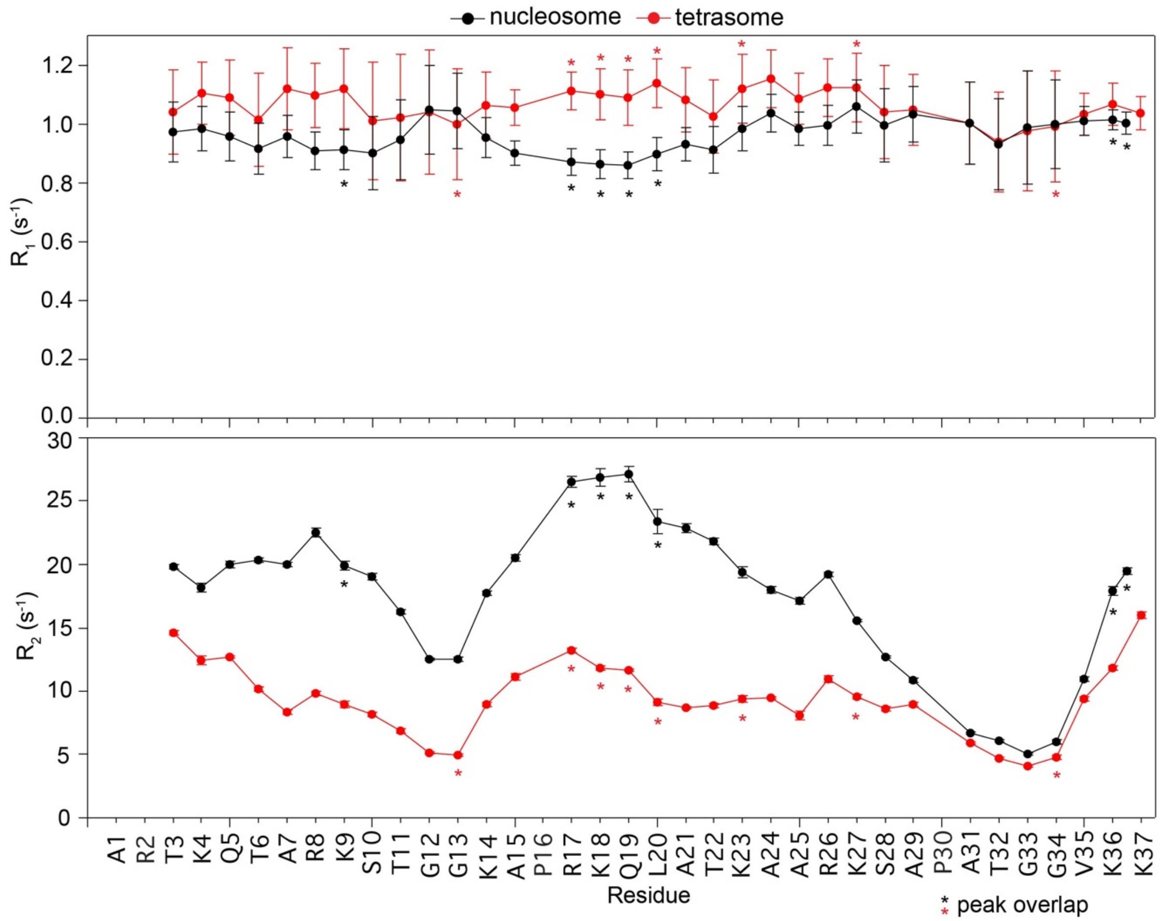

Supplementary Figure S8. H3 tail ^{15}N-relaxation rates (R_1, R_2) are plotted as a function of H3 tail residue for ^{15}N-H3 nucleosome (black) and tetrasome (red). Residues with peak overlap are marked by ‘*’.

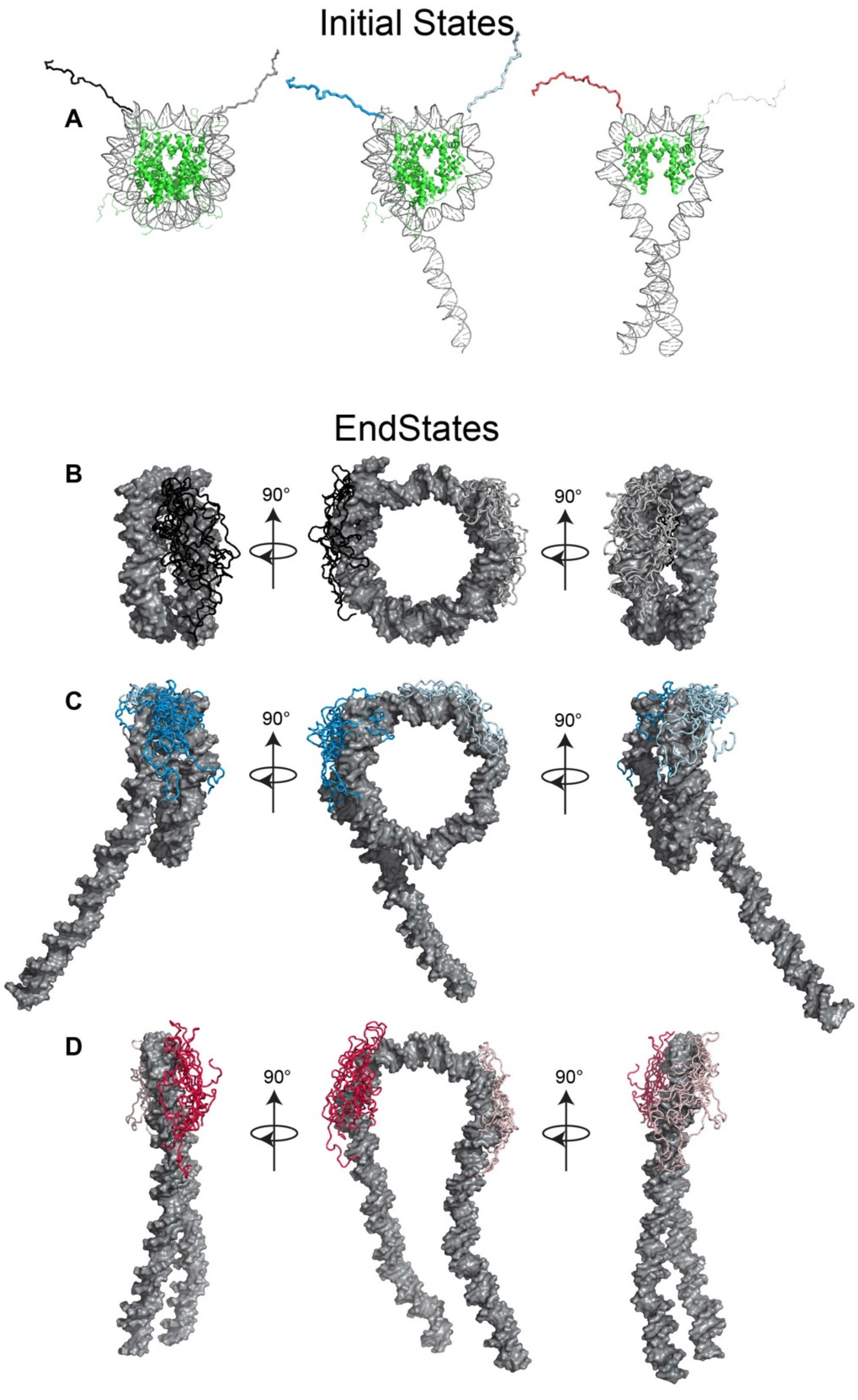

Supplementary Figure S9. (A) Starting states of nucleosomes and subnucleosomes for MD simulations. Initial states are shown for each of nucleosome (left), hexasome (middle), and tetrasome (right). **B-D.** End states of the H3 tails from ten simulations are shown on DNA from a single simulation for each of nucleosome **(B)**, hexasome **(C)**, and tetrasome **(D)**. The core histones and other histone tails are present in the simulations but removed in the figure for ease of visualization. Tail1 or hex-N is in the darker shade of each color (black, blue, and red) while tail2 or hex-T is in the lighter shade.

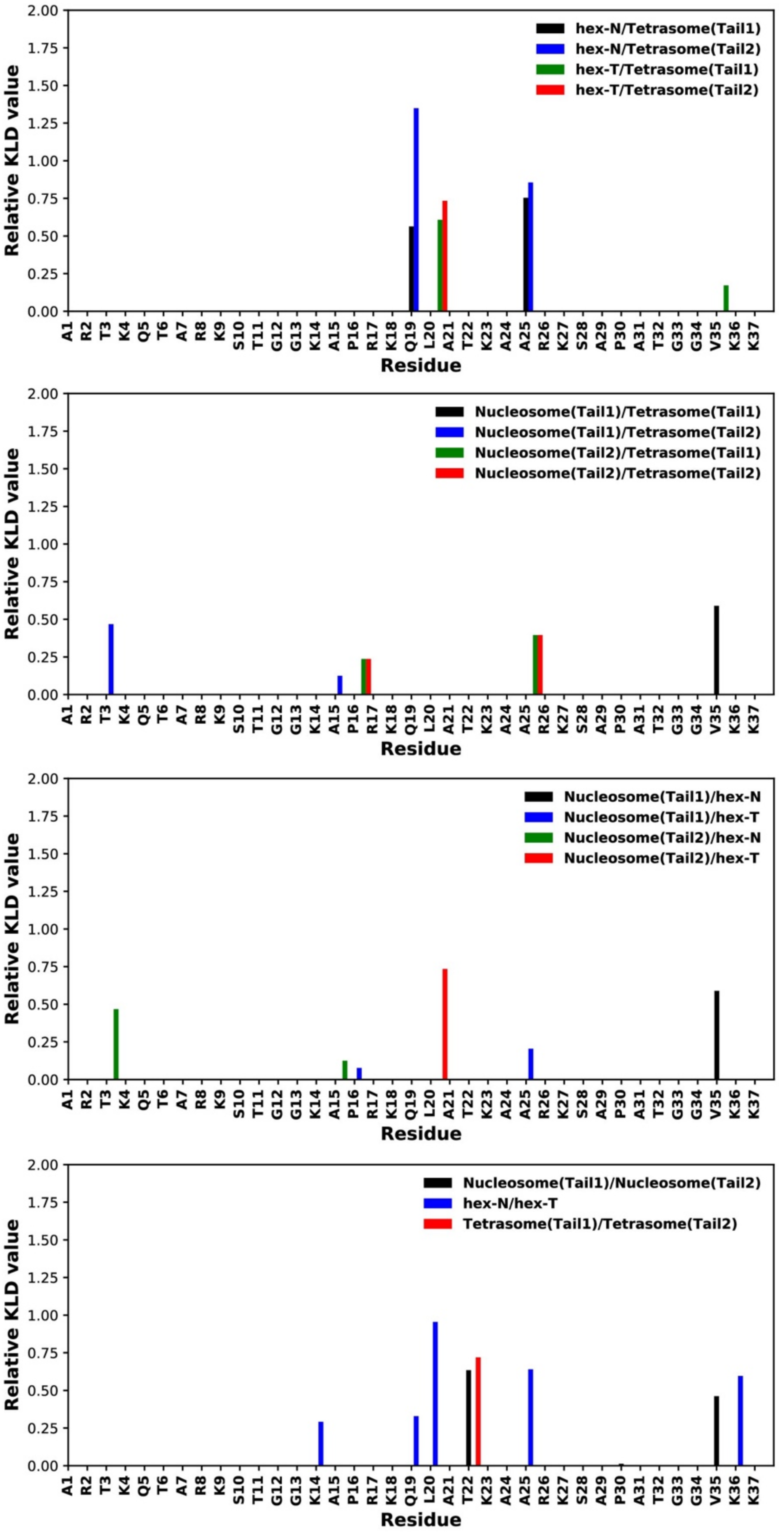

Supplementary Figure S10. Per residue Relative Kullback-Leibler divergence (KLD) of H3 tails. Plots are shown for different pair-wise comparisons of H3 tail ensembles, as labeled in the plot.

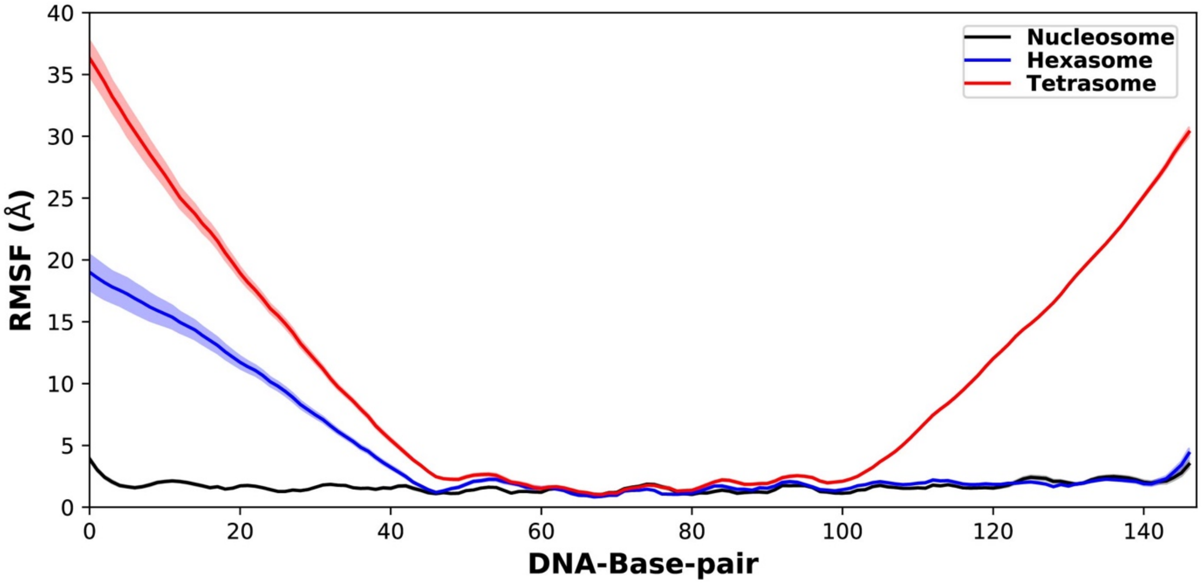

Supplementary Figure S11. DNA base-pair root mean square fluctuation (RMSF) values obtained from the equilibrated portion of MD trajectories. Plots are for nucleosome (black), hexasome (blue), and tetrasome (red) with the average of ten simulations plotted as a solid line and the standard error of the mean shaded. Data are plotted for all 147bp of DNA.

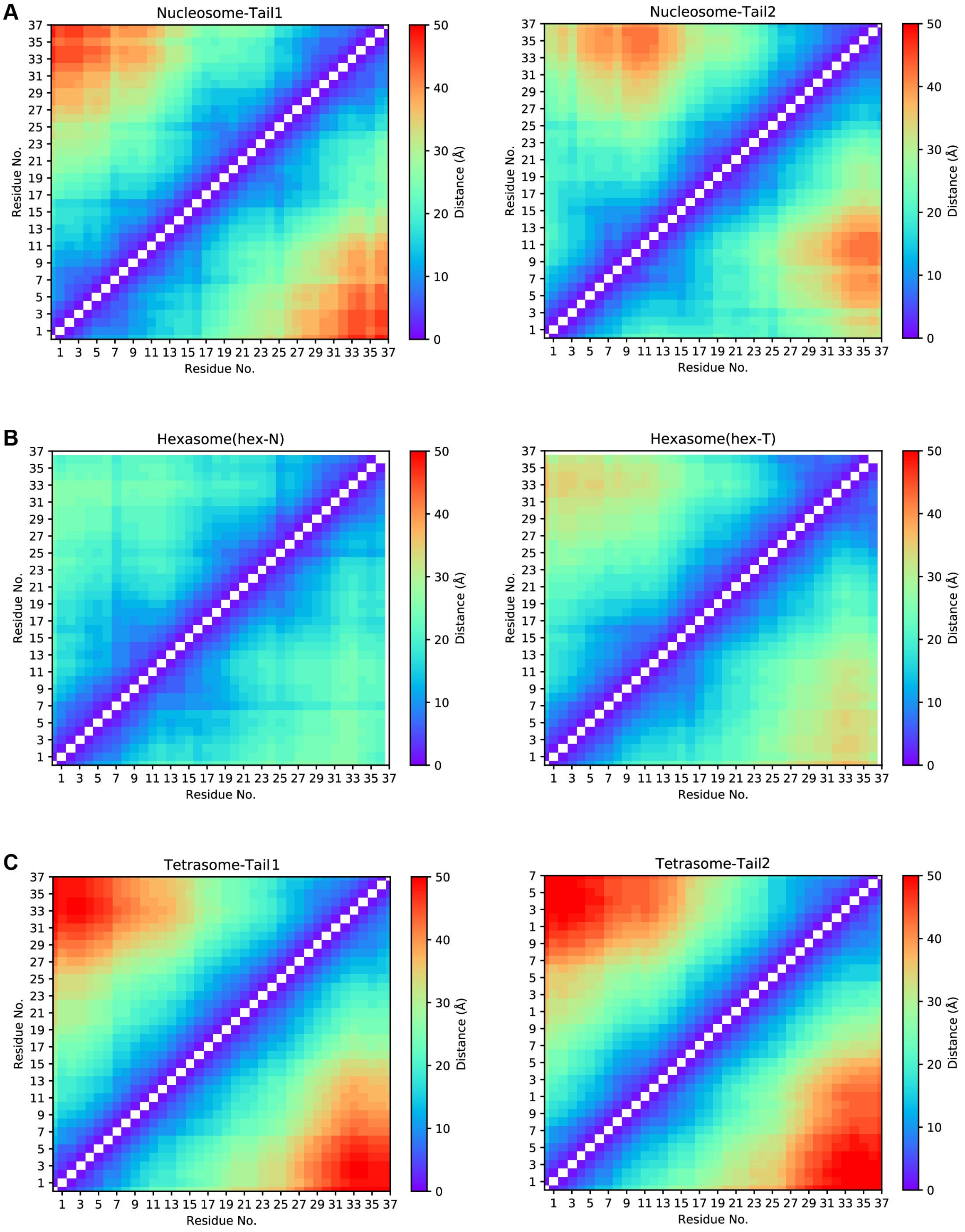

Supplementary Figure S12. Calculated average intra-tail distances along the H3 tails from MD simulations. Plots are shown for each of **(A)** nucleosome, **(B)** hexasome, and **(C)** tetrasome. These plots report on compactness of the H3 tails.

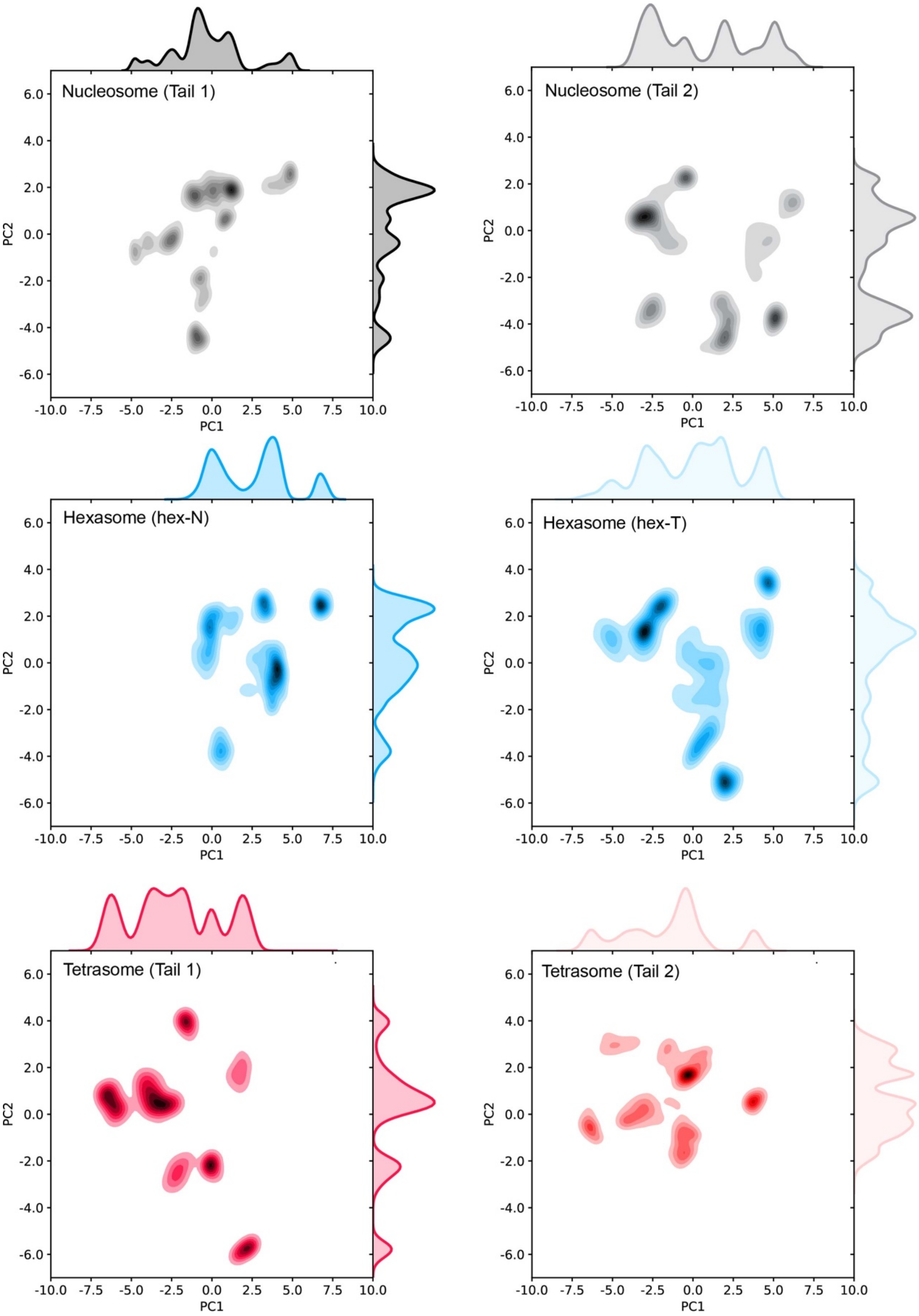

Supplementary Figure S13. Principal component analysis (PCA) of H3 tails (each aligned to itself). Two dimensional Density plots of PC1 and PC2 calculated for (top) nucleosome, (middle), hexasome and (bottom) tetrasome.

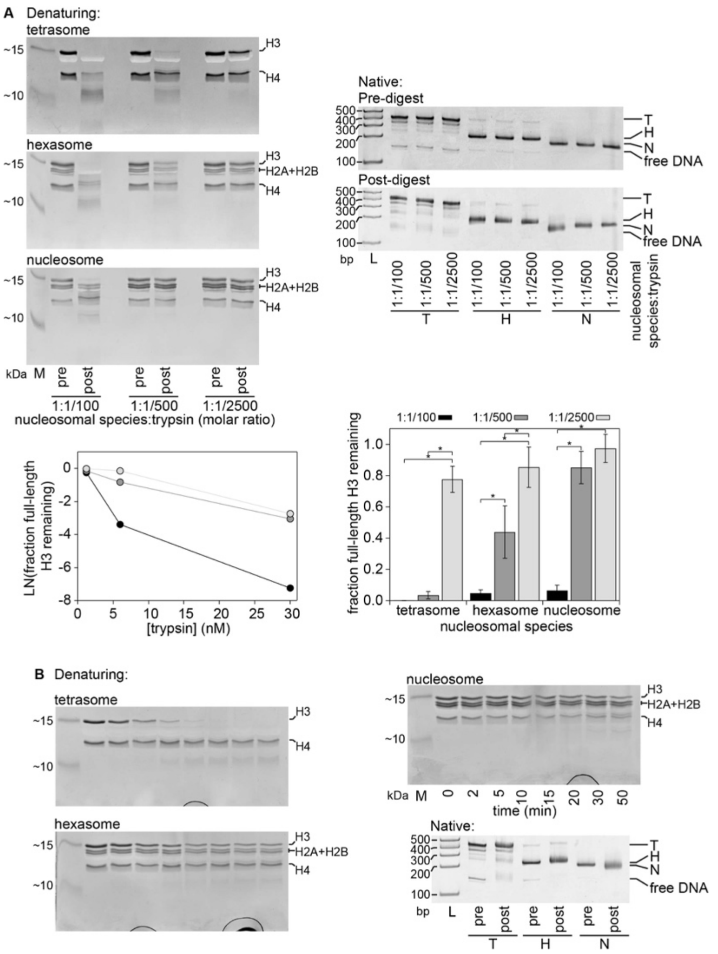

Supplementary Figure S14. Trypsin digestion assays to probe tail accessibility. **(A)** 18% denaturing acrylamide gels (left) were used to assess the progression of trypsin proteolysis. Gel samples were taken before the addition of trypsin (t=0, “pre”) and 20 minutes after the addition of trypsin (t=20min, “post”) for three different molar ratios of nucleosomal species:trypsin and a fixed concentration of the given nucleosomal species (3μM). Quantification of the gels are shown in **Figure 5A**. 5% native acrylamide gels (right) confirm that the nucleosomal species remain intact during the assay. The amount of free DNA does not appear to increase significantly while the band for each nucleosomal species remains intact but appears to blur as a result of the tail cleavage. An alternative representation of **Figure 5A** (lower right) highlights differences between ratios of trypsin for a given nucleosomal species that are statistically significant as determined by a two-way ANOVA followed by a tukey post-hoc analysis (*, p<0.05). Plotting the natural log of the fraction of full length H3 remaining as a function of trypsin concentration (lower left) suggests a linear relationship between k_{obs} and enzyme concentration (see Materials and Methods for more details). **(B)** 18% denaturing acrylamide gels (left) were used to assess the progression of trypsin proteolysis as a function of time at 3μM of a given nucleosomal species and a 1:1/500 molar ratio of trypsin. Gel samples were taken before the addition of trypsin (t=0) and at the indicated timepoints after the addition of trypsin. Quantification of the gels are shown in **Figure 5B**. 5% native acrylamide gels (lower right) confirm that the nucleosomal species remain intact during the assay. In the denaturing gels, the full-length position of each histone is labeled to the right of the gel. The denaturing gels were stained with Coomassie and include Spectra BR marker (M) for size reference while the native gels were visualized with ethidium bromide and include TrackIt 100bp DNA ladder (L) for size reference.

Supplementary Table S1. Summary of assigned chemical shifts for nucleosome, hexasome, and tetrasome. See also Fig S2. Due to the repetitive and unstructured nature of the H3 tail, there is chemical shift degeneracy in some of the resonances. Assignment uncertainty is noted in this table. For hexasome, the majority of peaks had degeneracy in C_α and C_β chemical shifts and thus could not be definitively assigned to one of the two copies of H3.
